# Childhood Loneliness and Cognitive Decline and Dementia Risk in Middle-Aged and Older Adults

**DOI:** 10.1001/jamanetworkopen.2025.31493

**Published:** 2025-09-12

**Authors:** Jinqi Wang, Danyang Jiao, Xiaoyu Zhao, Yixing Tian, Haibin Li, Xia Li, Chen Sheng, Lixin Tao, Hui Chen, Zhiyuan Wu, Xiuhua Guo

**Affiliations:** 1 Beijing Key Laboratory of Environment and Aging, Department of Epidemiology and Health Statistics, School of Public Health, Capital Medical University, Beijing, China; 2 Department of Cardiac Surgery, Heart Center and Beijing Key Laboratory of Hypertension, Beijing Chaoyang Hospital, Capital Medical University, Beijing, China; 3 Department of Mathematics and Statistics, La Trobe University, Melbourne, Australia; 4 Department of Epidemiology, Boston University School of Public Health, Boston, Massachusetts; 5 Department of Neurology, The First Affiliated Hospital, School of Medicine, Zhejiang University, Hangzhou, China; 6 School of Public Health, Second Affiliated Hospital, Zhejiang University School of Medicine, Hangzhou, China; 7 Department of Nutrition, Harvard T.H. Chan School of Public Health, Boston, Massachusetts; 8 National Institute for Data Science in Health and Medicine, Capital Medical University, Beijing, China; 9 School of Medical and Health Sciences, Edith Cowan University, Perth, Australia

## Abstract

**Importance:**

Adult loneliness has been linked to cognitive decline and dementia risk, but the long-term implications of childhood loneliness remain underexplored.

**Objectives:**

To investigate whether childhood loneliness is associated with cognitive decline and dementia risk and whether adult loneliness mediates or modifies these associations.

**Design, Setting, and Participants:**

This cohort study used data from the China Health and Retirement Longitudinal Study, a nationally representative cohort study conducted between June 1, 2011, and December 31, 2018, with a maximum follow-up of 7 years. Data analyses were performed from October 1, 2024, to January 15, 2025.

**Exposure:**

Childhood loneliness was defined as self-reported frequent feelings of loneliness and the absence of close friendships before age 17 years. Adult loneliness was measured by a single item from the Centre for Epidemiological Studies Depression Scale.

**Main Outcomes and Measures:**

Cognitive function was assessed through measures of episodic memory and executive function. Dementia was identified as the coexistence of cognitive and functional impairments or a self- or caregiver-reported physician diagnosis of dementia. Associations between childhood loneliness and cognitive decline were evaluated using linear mixed-effects models, and dementia risk was analyzed using Cox proportional hazards regression models.

**Results:**

Among 13 592 participants (mean [SD] age, 58.34 [9.39] years; 7175 [52.8%] female), 6525 (48.0%) experienced possible childhood loneliness, and 565 (4.2%) reported childhood loneliness. Compared with no childhood loneliness, childhood loneliness was associated with significantly faster cognitive decline (β, −0.03 [95% CI, −0.05 to −0.02] SD per year), as was possible childhood loneliness (β, −0.02 [95% CI, −0.02 to −0.01] SD per year). Moreover, childhood loneliness was associated with an increased risk of dementia (hazard ratio, 1.41 [95% CI, 1.03 to 1.93]). These associations remained significant when adjusted for adult loneliness and restricted to participants without adult loneliness. Adult loneliness mediated 8.5% (95% CI, 2.9% to 14.1%) of the association of childhood loneliness with cognitive decline and 17.2% (95% CI, 4.9%-29.5%) of the association with dementia risk but did not significantly modify these associations.

**Conclusions and Relevance:**

In this cohort study, childhood loneliness was associated with cognitive decline and dementia risk in middle and later adulthood, even in the absence of adult loneliness. Early interventions aimed at reducing childhood loneliness may help promote lifelong cognitive health and reducing dementia risk.

## Key Points

**Question:**

Is childhood loneliness associated with cognitive decline and incident dementia?

**Findings:**

In this cohort study of 13 592 participants, childhood loneliness was associated with faster cognitive decline and higher risk of incident dementia in middle and later adulthood. Adult loneliness was associated with mediating 8.5% of the association with cognitive decline and 17.2% of the association with dementia risk but did not significantly modify these associations.

**Meaning:**

These findings suggest that childhood loneliness may serve as an independent risk factor for later-life cognitive decline and dementia, highlighting the need for early interventions to mitigate its long-term implications for cognitive health throughout the life course.

## Teaser

This cohort study examines whether childhood loneliness is associated with cognitive decline and dementia risk in midlife and older age and whether adult loneliness mediates or modifies these associations.

## Introduction

Dementia is a worldwide public health concern,^1,2,3^ with the number of prevalent cases projected to increase from 50 million to 152 million by 2050.^3^ Given the current lack of effective treatments to modify the progression of dementia, identifying early, modifiable risk factors for cognitive decline and dementia is crucial for developing preventive strategies to mitigate this increasing burden of disease.^4,5^

Loneliness, a subjective and distressing experience resulting from the discrepancy between one’s perceived and desired levels of social connection, has been increasingly associated with various adverse health outcomes.^6,7^ Previous studies have linked midlife or late-life loneliness to a higher risk of cognitive decline and dementia.^8,9,10,11,12,13,14^ Importantly, loneliness is not confined to adulthood and can emerge in childhood. Globally, the prevalence of childhood loneliness has reached 13.2%.^15^ Nevertheless, the long-term consequences of childhood loneliness remain poorly understood. Early life is a critical stage for neurodevelopment, marked by heightened plasticity in neural and physiological systems, and children are particularly vulnerable to psychosocial stressors.^16^ While research has shown that adverse childhood experiences can lead to permanent changes in brain structure and function,^16,17^ the specific associations of childhood loneliness with later-life cognitive decline and dementia remain underexplored.

Additionally, early-life psychosocial stressors may increase the risk of loneliness in adulthood,^18^ which may act as a potential mediator and in turn contribute to poorer cognitive health.^19^ Meanwhile, favorable psychosocial factors in adulthood may buffer against the adverse cognitive consequences of early-life adversity.^20^ However, the potential mediating and moderating roles of adult loneliness in the associations between childhood loneliness and cognitive outcomes remain unclear.

To address these research gaps, we used nationally representative data from the China Health and Retirement Longitudinal Study (CHARLS) to investigate the long-term associations of childhood loneliness with cognitive decline and dementia in middle and later adulthood. Additionally, we explored whether adult loneliness modified or mediated these associations.

## Methods

The CHARLS protocols were approved by the ethics review committee of Peking University, and this analysis used deidentified data from a publicly available source; therefore, no additional ethics approval was required. All participants provided written informed consent. This study adhered to the Strengthening the Reporting of Observational Studies in Epidemiology (STROBE) reporting guideline.

### Study Population

CHARLS is a nationwide cohort study of Chinese adults aged 45 years and older. A total of 17 707 participants from 150 county-level units across 28 provinces were recruited in 2011 and followed-up in 2013, 2015, 2018, and 2020.^21,22^ Information on early-life experiences before age 17 years, including the assessment of childhood loneliness, was collected through face-to-face interviews during the 2014 Life History Survey.^23^

We restricted our analysis to CHARLS waves conducted before the COVID-19 pandemic (ie, 2011-2018) to avoid pandemic-related bias.^24,25,26^ Based on inclusion and exclusion criteria, 13 592 participants were included in the analyses of childhood loneliness and dementia risk, and 10 880 participants were included in the analyses of cognitive decline. Participants with missing adult loneliness assessments were excluded from adult loneliness–related analyses (eMethods and eFigure 1 in Supplement 1).

### Assessment of Childhood and Adult Loneliness

We evaluated childhood loneliness in CHARLS using 2 key questions, aligning with the framework used in the UK Biobank^27,28^: (1) “When you were a child, how often did you feel lonely because you had no friends? Was it often, sometimes, not very often, or never?” and (2) “When you were a child, did you have a close friend?” Participants who reported both often feeling lonely and not having a close friend were defined as having childhood loneliness, those meeting only 1 criterion were defined as possible childhood loneliness, and individuals reporting neither were defined as no childhood loneliness. Adult loneliness was assessed using a single item from the 10-item Center for Epidemiological Studies Depression Scale: “In the past week, how often did you feel lonely?” consistent with prior studies.^8,14,28,29^ Detailed definitions are presented in the eMethods in Supplement 1.

### Assessment of Cognitive Function

Cognitive function was evaluated in 2 domains: episodic memory (range, 0-10 points; higher score indicates better memory) and executive function (range, 0-11 points; higher score indicates better function).^5,30^ The global cognitive score was calculated as the sum of the episodic memory and executive function scores, ranging from 0 to 21 (eMethods in Supplement 1). To facilitate direct comparison of cognitive decline across tests, cognitive scores were standardized to *Z*-scores by subtracting the mean and dividing by the SD of baseline scores.^20^

### Determination of Dementia

In accordance with prior studies,^31,32,33^ dementia was defined using an algorithmic case definition based on the coexistence of cognitive impairment and functional impairment, or a report from the participant or caregiver of a physician’s diagnosis of dementia or related diseases. Detailed diagnostic procedures are provided in the eMethods in Supplement 1.

### Covariates

Based on the directed acyclic graph (eFigure 2 in Supplement 1), covariates in the minimally sufficient adjustment set included baseline age, sex, educational level, childhood area of residence, and childhood socioeconomic position. Details of covariate assessment are described in the eMethods in Supplement 1.

### Statistical Analysis

We presented baseline characteristics by childhood loneliness status. Proportions of missing covariate data are shown in eTable 1 in Supplement 1. We applied multiple imputations with chained equations (5 imputations) to handle missing covariate data.

We used linear mixed-effects models with random intercepts and random slopes to estimate the mean differences and 95% CIs in cognitive decline rates by childhood loneliness status, with no childhood loneliness as the reference. Fixed-effects terms included childhood loneliness status, time, the interaction between childhood loneliness and time, and other covariates. Cox proportional hazards regression models, with age as the timescale and left truncation by age at study entry, were used to estimate the hazard ratios (HRs) and 95% CIs for incident dementia associated with childhood loneliness. The proportional hazards assumption was verified using Schoenfeld residuals tests, with no violations detected. We conducted 2 models: model 1 adjusted for the minimally sufficient set of covariates identified by directed acyclic graph, and model 2 further adjusted for adult loneliness to determine whether the observed associations were independent of loneliness experienced in adulthood. Additionally, we examined the independent associations of 2 items defining childhood loneliness (often feel lonely; no close friendship) with the study outcomes, mutually adjusted for each other.

To examine whether adult loneliness modified the associations, we performed stratified analysis by adult loneliness and performed 3-way and 2-way interaction tests for the 2 study outcomes, respectively.^20^ Additionally, we conducted causal mediation analyses to assess whether adult loneliness mediated the associations. Finally, we examined the joint association of childhood and adult loneliness by constructing a 6-level variable combining loneliness status at both life stages (eFigure 3 in Supplement 1). Further details on statistical methods are provided in (eMethods in Supplement 1). Multiple sensitivity analyses were conducted to assess the robustness of our findings (eMethods and eFigure 4 in Supplement 1).

Statistical significance was set at 2-sided *P* < .05. Statistical analyses were conducted using R software version 4.1.0 (R Project for Statistical Computing), SAS software version 9.4 (SAS Institute), and Stata software version 16 (StataCorp). Data analyses were performed from October 1, 2024, to January 15, 2025.

## Results

### Population Characteristics

Of 13 592 eligible participants (mean [SD] age, 58.34 [9.39] years; 7175 [52.8%] female), 6525 (48.0%) reported possible childhood loneliness, and 565 (4.2%) reported experiencing childhood loneliness. For the individual items, 883 participants (6.5%) reported often feeling lonely and 6772 participants (49.8%) reported having no close friends during childhood. Compared with participants without childhood loneliness, those experiencing childhood loneliness were older and more likely to have lived in rural areas during childhood, lower educational attainment and childhood socioeconomic status, and higher rates of adult loneliness and poorer cognition (Table 1). Baseline characteristics of included vs excluded participants are presented in eTable 2 and eTable 3 in Supplement 1.

**Table 1.** Study Sample Characteristics Grouped by Childhood Loneliness Status

Characteristic^a^	Participants, No. (%)				*P* value^b^
	Overall (n = 13 592)	No childhood loneliness (n = 6502)	Possible childhood loneliness (n = 6525)	Childhood loneliness (n = 565)	
Age, mean (SD), y	58.34 (9.39)	56.80 (9.24)	59.62 (9.28)	61.26 (9.48)	<.001
Sex					
Male	6417 (47.2)	3039 (46.7)	3102 (47.5)	276 (48.8)	.48
Female	7175 (52.8)	3463 (53.3)	3423 (52.5)	289 (51.2)	
Childhood area of residence					
Rural	12361 (91.4)	5718 (88.3)	6103 (94.0)	540 (95.9)	<.001
Urban	1167 (8.6)	754 (11.7)	390 (6.0)	23 (4.1)	
Educational level					
No formal education	6158 (45.4)	2328 (35.9)	3438 (52.8)	392 (69.4)	<.001
≤Junior high school	5860 (43.2)	3106 (47.9)	2600 (39.9)	154 (27.3)	
≥High school	1549 (11.4)	1055 (16.3)	475 (7.3)	19 (3.4)	
Childhood socioeconomic position					
High	1219 (9.0)	763 (11.8)	436 (6.7)	20 (3.6)	<.001
Medium	6925 (51.1)	3429 (52.8)	3340 (51.4)	156 (27.8)	
Low	5405 (39.9)	2299 (35.4)	2720 (41.9)	386 (68.7)	
Adult loneliness status					
No	8976 (71.0)	4460 (73.6)	4219 (69.7)	297 (56.8)	<.001
Yes	3661 (29.0)	1600 (26.4)	1835 (30.3)	226 (43.2)	
Depression score, mean (SD)	7.34 (5.87)	6.79 (5.68)	7.66 (5.93)	9.88 (6.45)	<.001
Smoking status					
Current	4259 (31.5)	1974 (30.5)	2110 (32.5)	175 (31.1)	.03
Former	1080 (8.0)	508 (7.9)	513 (7.9)	59 (10.5)	
Never	8184 (60.5)	3983 (61.6)	3872 (59.6)	329 (58.4)	
Drinking status					
>Once a month	3458 (25.6)	1678 (26.0)	1657 (25.5)	123 (21.9)	.08
≤Once a month	1053 (7.8)	530 (8.2)	483 (7.4)	40 (7.1)	
None of these	9009 (66.6)	4256 (65.8)	4354 (67.0)	399 (71.0)	
Sleep duration, h/d					
<7	6384 (50.2)	2992 (49.0)	3092 (50.8)	300 (56.1)	<.001
7-8	5297 (41.6)	2672 (43.8)	2440 (40.1)	185 (34.6)	
>8	1042 (8.2)	438 (7.2)	554 (9.1)	50 (9.3)	
Chronic diseases					
Heart disease	1525 (11.3)	739 (11.5)	722 (11.2)	64 (11.6)	.84
Stroke	223 (1.7)	99 (1.5)	112 (1.7)	12 (2.1)	.45
Cancer	125 (0.9)	55 (0.9)	66 (1.0)	4 (0.7)	.54
Diabetes	721 (5.4)	351 (5.5)	346 (5.4)	24 (4.3)	.50
Global cognitive *z*-score, mean (SD)^c^	0.00 (1.00)	0.18 (0.96)	−0.15 (1.01)	−0.40 (1.04)	<.001
Episodic memory *z*-score, mean (SD)^c^	0.00 (1.00)	0.14 (1.00)	−0.13 (0.98)	−0.21 (0.99)	<.001
Executive function *z*-score, mean (SD)^c^	0.00 (1.00)	0.15 (0.94)	−0.13 (1.03)	−0.41 (1.08)	<.001
Incident dementia	697 (5.1)	313 (4.8)	336 (5.1)	48 (8.5)	.001

^a^ Percentages may not total 100% because of rounding. Counts may not sum to column totals because of missing data. Missing data for covariates: age (n = 1), childhood area of residence (n = 64), educational level (n = 25), childhood socioeconomic position (n = 43), adult loneliness (n = 955), depression score (n = 56), smoking status (n = 69), drinking status (n = 72), sleep duration (n = 869), heart disease (n = 138), stroke (n = 97), cancer (n = 125), and diabetes (n = 188).

^b^ Continuous variables were compared using 1-way analysis of variance test or Kruskal-Wallis test. Categorical variables were compared using χ^2^ test or Fisher exact test.

^c^ Assessed among 10 880 participants.

### Childhood Loneliness and Cognitive Decline

The associations between childhood loneliness and adult cognitive decline are summarized in Table 2 and eTable 4 in Supplement 1. After adjusting for potential confounding factors in model 1, childhood loneliness (vs no childhood loneliness) was associated with lower baseline global cognitive *Z*-scores (β, −0.14 [95% CI, −0.21 to −0.06] SD). Moreover, compared with participants without childhood loneliness (mean rate, −0.03 [95% CI, −0.03 to −0.02] SD per year), childhood loneliness was associated with an additional 0.03 (95% CI, 0.02 to 0.05) SD per year faster global cognitive decline. Those with possible childhood loneliness also had lower baseline global cognitive scores (β, −0.08 [95% CI, −0.11 to −0.05] SD) and experienced accelerated global cognitive decline (β, −0.02 [95% CI, −0.02 to −0.01] SD per year). The findings were similar by domain-specific cognitive tests and remained significant after adjusting for adult loneliness (model 2). Both childhood loneliness items (often feeling lonely and having no close friends) were independently significantly associated with faster cognitive decline (eTable 5 in Supplement 1).

**Table 2.** Associations Between Childhood Loneliness and Cognitive Decline During Follow-Up in the Overall Population

Variable	Global cognitive function		Episodic memory		Executive function	
	β (95% CI)^a^	*P* value	β (95% CI)^a^	*P* value	β (95% CI)^a^	*P* value
**Model 1^b^**						
Childhood loneliness status^c^						
No childhood loneliness	0 [Reference]	NA	0 [Reference]	NA	0 [Reference]	NA
Possible childhood loneliness	−0.08 (−0.11 to −0.05)	<.001	−0.05 (−0.09 to −0.02)	.002	−0.07 (−0.10 to −0.04)	<.001
Childhood loneliness	−0.14 (−0.21 to −0.06)	<.001	0.03 (−0.06 to 0.11)	.52	−0.19 (−0.27 to −0.11)	<.001
Time^d^	−0.03 (−0.03 to −0.02)	<.001	0.00 (0.00 to 0.01)	.52	−0.04 (−0.04 to −0.03)	<.001
Childhood loneliness status × time^d^						
No childhood loneliness	0 [Reference]	NA	0 [Reference]	NA	0 [Reference]	NA
Possible childhood loneliness	−0.02 (−0.02 to −0.01)	<.001	−0.02 (−0.03 to −0.02)	<.001	−0.01 (−0.01 to 0.00)	.005
Childhood loneliness	−0.03 (−0.05 to −0.02)	<.001	−0.05 (−0.07 to −0.03)	<.001	−0.01 (−0.03 to 0.00)	.06
**Model 2^e^**						
Childhood loneliness status^c^						
No childhood loneliness	0 [Reference]	NA	0 [Reference]	NA	0 [Reference]	NA
Possible childhood loneliness	−0.07 (−0.11 to −0.04)	<.001	−0.05 (−0.08 to −0.02)	.003	−0.07 (−0.10 to −0.04)	<.001
Childhood loneliness	−0.11 (−0.19 to −0.03)	.006	0.05 (−0.04 to 0.13)	.31	−0.16 (−0.24 to −0.09)	<.001
Time^d^	−0.03 (−0.03 to −0.02)	<.001	0.00 (0.00 to 0.01)	.50	−0.04 (−0.04 to −0.03)	<.001
Childhood loneliness status × time^d^						
No childhood loneliness	0 [Reference]	NA	0 [Reference]	NA	0 [Reference]	NA
Possible childhood loneliness	−0.02 (−0.02 to −0.01)	<.001	−0.02 (−0.03 to −0.02)	<.001	−0.01 (−0.01 to 0.00)	.004
Childhood loneliness	−0.03 (−0.05 to −0.02)	<.001	−0.05 (−0.07 to −0.03)	<.001	−0.01 (−0.03 to 0.00)	.06

Abbreviation: NA, not applicable.

^a^ The β coefficients were estimated using linear mixed-effects models.

^b^ Model 1 was adjusted for age, sex, educational level, childhood area of residence, and childhood socioeconomic position.

^c^ The β coefficient and its 95% CI are reported as SD.

^d^ The β coefficient and its 95% CI are reported as SD per year.

^e^ Model 2 was further adjusted for adult loneliness, which was included only among the 10 834 participants with available adult loneliness data. Missing data for other covariates were handled by multiple imputation with chained equations.

As shown in Table 3 and eTable 6 and eTable 7 in Supplement 1, no multiplicative interaction was observed between childhood and adult loneliness on cognitive decline, which was further confirmed by stratified analysis by adult loneliness status (adult nonloneliness group: β, −0.04 [95% CI, −0.06 to −0.02] SD per year; adult loneliness group: β, −0.03 [95% CI, −0.05 to 0.00] SD per year). The Figure shows the estimated cognitive decline trajectories by childhood loneliness status in the overall population, as well as subgroups stratified by adult loneliness. Mediation analyses indicated that adult loneliness mediated 2.3% (95% CI, 0.2% to 4.4%) and 8.5% (95% CI, 2.9% to 14.1%) of the associations of possible childhood loneliness and childhood loneliness, respectively, with cognitive decline (eTable 8 in Supplement 1).

**Table 3.** Associations Between Childhood Loneliness and Cognitive Decline During Follow-Up, Stratified by Adult Loneliness

Variable	Global cognitive function		Episodic memory		Executive function		
	β (95% CI)^a^	*P* value	β (95% CI)^a^	*P* value	β (95% CI)^a^	*P* value
**Adult nonloneliness group (n = 7840)**						
Childhood loneliness status^b^						
No childhood loneliness	0 [Reference]	NA	0 [Reference]	NA	0 [Reference]	NA
Possible childhood loneliness	−0.07 (−0.11 to −0.04)	<.001	−0.05 (−0.09 to −0.01)	.02	−0.06 (−0.10 to −0.03)	<.001
Childhood loneliness	−0.13 (−0.23 to −0.02)	.02	0.03 (−0.09 to 0.14)	.63	−0.18 (−0.28 to −0.08)	<.001
Time^c^	−0.03 (−0.03 to −0.02)	<.001	0.00 (0.00 to 0.01)	.25	−0.04 (−0.04 to −0.03)	<.001
Childhood loneliness status × time^c^						
No childhood loneliness	0 [Reference]	NA	0 [Reference]	NA	0 [Reference]	NA
Possible childhood loneliness	−0.02 (−0.02 to −0.01)	<.001	−0.02 (−0.03 to −0.01)	<.001	−0.01 (−0.02 to 0.00)	.004
Childhood loneliness	−0.04 (−0.06 to −0.02)	<.001	−0.05 (−0.08 to −0.03)	<.001	−0.02 (−0.03 to 0.00)	.10
**Adult loneliness group (n = 2994)**						
Childhood loneliness status^b^						
No childhood loneliness	0 [Reference]	NA	0 [Reference]	NA	0 [Reference]	NA
Possible childhood loneliness	−0.09 (−0.15 to −0.03)	.003	−0.06 (−0.12 to 0.00)	0.06	−0.08 (−0.14 to −0.02)	.01
Childhood loneliness	−0.09 (−0.22 to 0.04)	.16	0.05 (−0.08 to 0.18)	0.44	−0.14 (−0.27 to −0.01)	.03
Time^c^	−0.03 (−0.03 to −0.02)	<.001	0.00 (−0.01 to 0.01)	0.49	−0.03 (−0.04 to −0.02)	<.001
Childhood loneliness status × time^c^						
No childhood loneliness	0 [Reference]	NA	0 [Reference]	NA	0 [Reference]	NA
Possible childhood loneliness	−0.01 (−0.03 to 0.00)	.01	−0.02 (−0.04 to −0.01)	.002	−0.01 (−0.02 to 0.01)	.31
Childhood loneliness	−0.03 (−0.05 to 0.00)	.04	−0.04 (−0.08 to −0.01)	.01	−0.01 (−0.04 to 0.01)	.28

Abbreviation: NA, not applicable.

^a^ The β coefficients were estimated using linear mixed-effects models adjusted for age, sex, educational level, childhood area of residence, and childhood socioeconomic position. Missing covariate data were handled by multiple imputation with chained equations.

^b^ The β coefficient and its 95% CI are reported as SD.

^c^ The β coefficient and its 95% CI are reported as SD per year.

**Figure.**
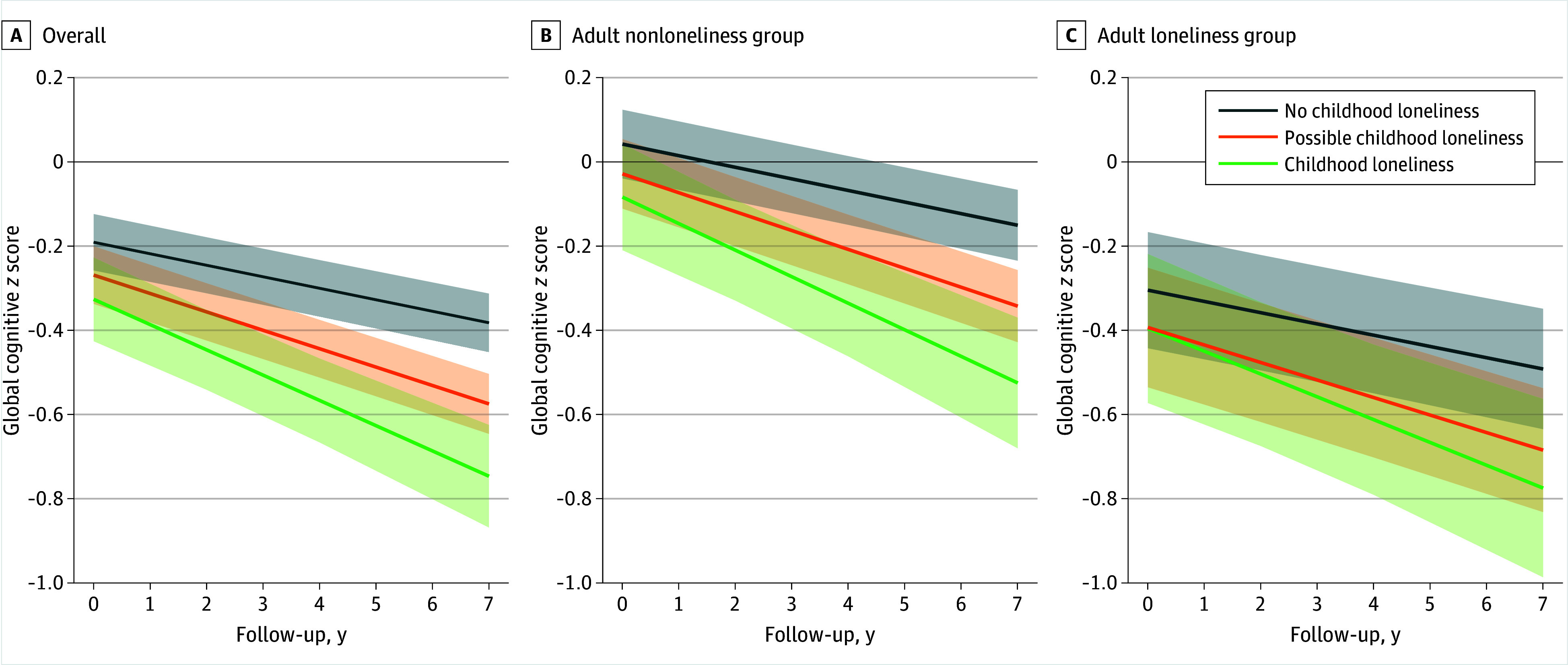
Estimated Trajectories of Global Cognition During Follow-Up by Childhood Loneliness Groups Using Linear Mixed-Effects Models Estimated global cognitive z-scores are based on models adjusted for age, sex, educational level, childhood area of residence, and childhood socioeconomic position (missing data for covariates were handled by multiple imputation with chained equations) by childhood loneliness status. The intercept of each line reflects the baseline cognitive *z*-scores, while the slope represents the annual rate of cognitive decline. Shading indicates 95% CI.

### Childhood Loneliness and Dementia Risk

During a median (IQR) follow-up of 7 (7-7) years, 697 participants (5.1%) developed incident dementia. Individuals with childhood loneliness (13.5 events per 1000 person-years) and possible childhood loneliness (8.0 events per 1000 person-years) were more likely to develop dementia than those without (7.5 events per 1000 person-years). In multivariable-adjusted model 1, compared with no childhood loneliness, we observed higher dementia risk for individuals with childhood loneliness (HR, 1.41 [95% CI, 1.03-1.93]), whereas for individuals with possible childhood loneliness, the difference was not statistically significant (HR, 0.91 [95% CI, 0.78-1.07]) (Table 4; eTable 9 in Supplement 1). Associations remained similar after adjustment for adult loneliness. For the individual items, reporting often feeling lonely in childhood was associated with higher dementia risk (HR, 1.51 [95% CI, 1.17-1.95]), whereas reporting having no close friends in childhood was not (HR, 0.94 [95% CI, 0.80-1.11]) (eTable 10 in Supplement 1).

**Table 4.** Associations Between Childhood Loneliness and Incident Dementia During Follow-Up, in the Overall Population and Stratified by Adult Loneliness

Variable	No.	Incidence rate per 1000 PY, No. (%)	Model 1		Model 2	
			HR (95% CI)^a^	*P* value	HR (95% CI)^b^	*P* value
**Overall (N = 13 592)**						
Childhood loneliness status						
No childhood loneliness	6502	313 (7.5)	1 [Reference]	NA	1 [Reference]	NA
Possible childhood loneliness	6525	336 (8.0)	0.91 (0.78-1.07)	.25	0.96 (0.81-1.13)	.61
Childhood loneliness	565	48 (13.5)	1.41 (1.03-1.93)	.03	1.46 (1.05-2.01)	.02
**Adult nonloneliness group (n = 8976)**						
Childhood loneliness status						
No childhood loneliness	4460	177 (6.1)	1 [Reference]	NA	NA	NA
Possible childhood loneliness	4219	181 (6.6)	0.91 (0.74-1.13)	.40	NA	NA
Childhood loneliness	297	24 (12.9)	1.62 (1.04-2.51)	.03	NA	NA
**Adult loneliness group (n = 3661)**						
Childhood loneliness status						
No childhood loneliness	1600	107 (10.5)	1 [Reference]	NA	NA	NA
Possible childhood loneliness	1835	134 (11.5)	1.04 (0.80-1.35)	.80	NA	NA
Childhood loneliness	226	22 (15.3)	1.38 (0.86-2.23)	.18	NA	NA

Abbreviations: HR, hazard ratio; NA, not applicable; PY, person-years.

^a^ Estimated using Cox proportional hazards regression models, with age as the timescale and left truncation by age at study entry. Model 1 was adjusted for age (time scale), sex, educational level, childhood area of residence, and childhood socioeconomic position.

^b^ Model 2 was further adjusted for adult loneliness, which was included only among the 12 637 participants with available adult loneliness data. Missing data for other covariates were handled by multiple imputation with chained equations.

The associations between childhood loneliness and dementia risk did not significantly differ by adult loneliness (Table 4; eTable 11 in Supplement 1). The risk for childhood loneliness was higher among participants without adult loneliness (HR, 1.62 [95% CI, 1.04-2.51]), but not among those with adult loneliness (HR, 1.38 [95% CI, 0.86-2.23]). Adult loneliness mediated 17.2% (95% CI, 4.9%-29.5%) of the association between childhood loneliness and incident dementia (eTable 8 in Supplement 1).

### Joint Analysis of Childhood and Adult Loneliness

Compared with participants with neither childhood nor adult loneliness, those with loneliness only in childhood had the fastest cognitive decline (β, −0.04 [95% CI, −0.06 to −0.02] SD per year), followed by those with loneliness in both childhood and adulthood (β, −0.03 [95% CI, −0.05 to 0.00] SD per year) (eTable 12 in Supplement 1). However, loneliness in adulthood alone was not significantly associated with the rate of cognitive decline (β, 0.00 [95% CI, −0.01 to 0.01] SD per year). Furthermore, individuals with both childhood and adult loneliness had the highest dementia risk (HR, 2.05 [95% CI, 1.30 to 3.22]), followed by those with childhood loneliness only (HR, 1.71 [95% CI, 1.11 to 2.64]), and then those with only adult loneliness (HR, 1.63 [95% CI, 1.28 to 2.08]).

### Sensitivity Analyses

Findings remained robust after restricting analyses to participants with complete covariate data; adjusting for chronic conditions, depression, and healthy lifestyles; and excluding participants with baseline cognitive impairment (eTables 13-18 in Supplement 1). In more nuanced analyses of childhood loneliness frequency, participants reporting frequent loneliness experienced the most rapid cognitive decline and the highest dementia risk (eTable 19 and eTable 20 in Supplement 1). Moreover, in mixed-effects Tobit models accounting for ceiling effects, childhood loneliness remained significantly associated with executive function (eTable 21 in Supplement 1). Finally, a significant interaction between childhood loneliness status and childhood area of residence was found for dementia risk, whereas other interactions were not significant (eFigure 5 in Supplement 1).

## Discussion

In this large-scale, nationally representative longitudinal cohort study, childhood loneliness was significantly associated with accelerated cognitive decline and higher dementia risk in middle and later adulthood. The associations were only partially explained by adult loneliness and persisted even among individuals who did not experience loneliness in adulthood. These findings highlight that childhood loneliness may have enduring implications for cognitive health and dementia risk across the lifespan.

There is evolving evidence suggesting loneliness as a risk factor for cognitive decline and dementia.^8,9,10,11,12,13,14^ However, existing studies have focused primarily on adult loneliness, while the role of childhood loneliness has rarely been evaluated. A 2025 study reported no association between early-life loneliness and cognitive function in early adulthood (mean age of cognitive assessment, 26.4 years).^34^ However, adverse cognitive outcomes typically emerge in later life,^35^ thus the long-term cognitive impacts of early-life loneliness remained unclear. Based on this, we examined this association in middle-aged and older adults and provided novel evidence that childhood loneliness was associated with later-life cognitive function and dementia risk, even when controlling for childhood sociodemographic characteristics, psychological factors, lifestyle behaviors, and chronic conditions in adulthood.

Across the life course, loneliness may be transient for some people but persistent for others.^14^ Several studies have examined associations of transitions in loneliness during midlife or later with health outcomes, demonstrating that persistent loneliness in midlife is associated with increased dementia risk, cognitive decline, and brain atrophy, whereas recovery from loneliness is not associated with higher risks of these adverse outcomes.^8,14,36^ Our study broadened the perspective to the entire life course and found that individuals who experienced childhood loneliness, even in the absence of adult loneliness, still exhibited accelerated cognitive decline and elevated dementia risk. Adult loneliness only partially mediated the association of childhood loneliness with later-life cognitive decline and dementia risk and did not modify the strength of these associations. These findings could be explained by the developmental origins of health and disease hypothesis,^37^ which posits that the developing brain and nervous system are particularly vulnerable to adverse exposures and highly plastic,^38^ allowing childhood loneliness to induce lasting effects on brain health.^16,17,39^ Later-life experiences may not fully compensate for these early impacts. Notably, individuals with loneliness only in childhood exhibited faster cognitive decline and a higher dementia risk than those with loneliness exclusively in adulthood, highlighting childhood as a critical period for loneliness intervention.

The exact mechanisms underlying the association of childhood loneliness with cognitive decline and dementia remain unclear. Childhood is a critical period for the formation of health-related behaviors and psychological patterns that often persist into adulthood.^34^ Children experiencing loneliness often adopt unhealthy behaviors as coping mechanisms to alleviate emotional distress.^35^ Prior studies have found associations between childhood loneliness and smoking, alcohol use, substance abuse, and sleep disorders.^35,40^ Moreover, childhood loneliness has been associated with adult psychiatric disorders.^7,34,41^ These behavioral and psychological factors may adversely affect neurodevelopment, which influences later-life cognitive performance.^42^ Additionally, the distress and low mood associated with childhood loneliness may impair peer interactions, which are critical for providing cognitive stimulation, promoting neuroplasticity, and building greater cognitive reserve during childhood, thereby hindering the development of a more efficient cognitive network.^43,44^ As for the physiological pathway, childhood loneliness may act as a chronic interpersonal stressor, triggering prolonged activation of the hypothalamic-pituitary-adrenal axis, elevated levels of stress hormones (eg, cortisol), hippocampal damage, overactivation of the sympathetic nervous system, oxidative stress, and immune system dysregulation.^44,45,46,47^ During a highly sensitive developmental window in childhood, these physiological disruptions may irreversibly alter brain structure and function, thereby increasing vulnerability to cognitive impairment and dementia later in life.^8,9,16,17^

Our findings have important implications for promoting later-life cognitive health and underscore the urgent need to identify, prevent, and address childhood loneliness through concerted efforts by families, schools, health care practitioners, and policymakers. Effective strategies may include increasing opportunities for social contact, promoting social skills development, enhancing social support networks, creating supportive environments in schools and communities, and providing mental health services targeting childhood loneliness.^7^ However, to date, no established guidelines or recommendations emphasize the importance of addressing childhood loneliness. Our study provides novel insights into this gap, highlighting the need for future research to explore the long-term benefits of interventions targeting childhood loneliness on cognitive health and overall well-being.

### Limitations

To our knowledge, this is the first study to assess the association of childhood loneliness with later-life cognitive function and incident dementia. Nevertheless, several limitations should be acknowledged. First, childhood loneliness was retrospectively assessed through self-report, which may be subject to recall bias, although previous studies have shown the reliability of such measures, and their associations with health outcomes align with findings from prospective-record data.^48,49,50^ Moreover, our findings are robust after restricting analyses to participants with relatively better cognitive performance at baseline to reduce recall bias. Despite this, there is a possibility of nondifferential misclassification that diluted the associations, so the magnitudes of the results should be interpreted with caution. Second, as there is no standardized measure of loneliness, we referenced prior studies to define childhood loneliness using friendship-related questions and adult loneliness using a single item from the 10-item Center for Epidemiological Studies Depression Scale. These simplified self-report measures may not fully capture the multidimensional nature of loneliness. Third, while our cognitive assessment had good consistency with the Mini-Mental State Examination and the Clinical Dementia Rating Scale,^51^ further investigation is warranted into the specific cognitive domains that could be impacted by childhood loneliness using more nuanced assessments. Fourth, our study focused exclusively on a Chinese population and participants excluded from analyses were likely less healthy, so future research should assess whether our findings generalize to populations with diverse sociocultural, environmental, and genetic backgrounds and varying health conditions. Fifth, given the observational nature of this study, unmeasured and residual confounding may exist, and the extent to which the observed associations reflect causal effects remains to be explored.

## Conclusions

In this cohort study, childhood loneliness was significantly associated with faster cognitive function decline and an increased risk of dementia in later life, even for those who no longer experienced loneliness in adulthood. Public health initiatives aimed at preventing and reducing loneliness should begin in early life to mitigate its long-term implications for cognitive health and well-being.

## Notes

Supplement 1.
eMethods.
**eFigure 1.** Flowchart of participant selection **eFigure 2.** Directed acyclic graph displaying the hypothetical confounding structure of the study **eFigure 3.** Transition of loneliness from childhood to adulthood **eFigure 4.** Distribution of global cognition, episodic memory, and executive function scores to assess ceiling effects **eFigure 5.** Associations of childhood loneliness with rate of cognitive decline and incident dementia across different subgroups **eTable 1.** Missing data proportions for covariates **eTable 2.** Baseline characteristics of participants included in the analyses of incident dementia versus those excluded **eTable 3.** Baseline characteristics of participants included in the analyses of cognitive decline versus those excluded **eTable 4.** Associations between childhood loneliness and cognitive decline during follow-up in the overall population, with complete regression output for all covariates **eTable 5.** Associations between two childhood loneliness items (often feel lonely in childhood; no close friends in childhood) and cognitive decline during follow-up **eTable 6.** Modifying role of adult loneliness in the association between childhood loneliness and cognitive decline during follow-up **eTable 7.** Associations between childhood loneliness and cognitive decline during follow-up stratified by adult loneliness, with complete regression output for all covariates **eTable 8.** Mediating role of adult loneliness in the association of childhood loneliness with cognitive decline and incident dementia **eTable 9.** Associations between childhood loneliness and incident dementia during follow-up in the overall population and stratified by adult loneliness, with complete regression output for all covariates **eTable 10.** Associations between two childhood loneliness items (often feel lonely in childhood; no close friends in childhood) and incident dementia during follow-up **eTable 11.** Modifying role of adult loneliness in the associations between childhood loneliness and incident dementia during follow-up **eTable 12.** Joint associations of childhood and adulthood loneliness with cognitive decline and dementia risk during follow-up **eTable 13.** Associations between childhood loneliness and cognitive decline during follow-up after excluding participants with missing data on covariates **eTable 14.** Associations between childhood loneliness and incident dementia during follow-up after excluding participants with missing data on covariates **eTable 15.** Associations between childhood loneliness and cognitive decline during follow-up after further adjustment for chronic diseases, depression, and healthy lifestyles **eTable 16.** Associations between childhood loneliness and incident dementia during follow-up after further adjustment for chronic diseases, depression, and healthy lifestyles **eTable 17.** Associations between childhood loneliness and cognitive decline during follow-up after excluding participants with cognitive impairment at baseline **eTable 18.** Associations between childhood loneliness and incident dementia during follow-up after excluding participants with cognitive impairment at baseline **eTable 19.** Associations between frequency of childhood loneliness and cognitive decline during follow-up **eTable 20.** Associations between frequency of childhood loneliness and incident dementia during follow-up **eTable 21.** Associations of childhood loneliness with executive function, assessed using mixed-effects Tobit models to account for ceiling effects
eReferences.

Supplement 2.
Data Sharing Statement
