# Supplementary material for: Childhood Loneliness and Cognitive Decline and Dementia Risk in Middle-Aged and Older Adults
Source: JAMA Netw Open. 2025 Sep 12;8(9):e2531493. doi: 10.1001/jamanetworkopen.2025.31493 (PMC12432641; doi:10.1001/jamanetworkopen.2025.31493)
Supplement: Supplement 1. — eMethods. eFigure 1. Flowchart of participant selection eFigure 2. Directed acyclic graph displaying the hypothetical confounding structure of the study eFigure 3. Transition of loneliness from childhood to adulthood eFigure 4. Distribution of global cognition, episodic memory, and executive function scores to assess ceiling effects eFigure 5. Associations of childhood loneliness with rate of cognitive decline and incident dementia across different subgroups eTable 1. Missing data proportions for covariates eTable 2. Baseline characteristics of participants included in the analyses of incident dementia versus those excluded eTable 3. Baseline characteristics of participants included in the analyses of cognitive decline versus those excluded eTable 4. Associations between childhood loneliness and cognitive decline during follow-up in the overall population, with complete regression output for all covariates eTable 5. Associations between two childhood loneliness items (often feel lonely in childhood; no close friends in childhood) and cognitive decline during follow-up eTable 6. Modifying role of adult loneliness in the association between childhood loneliness and cognitive decline during follow-up eTable 7. Associations between childhood loneliness and cognitive decline during follow-up stratified by adult loneliness, with complete regression output for all covariates eTable 8. Mediating role of adult loneliness in the association of childhood loneliness with cognitive decline and incident dementia eTable 9. Associations between childhood loneliness and incident dementia during follow-up in the overall population and stratified by adult loneliness, with complete regression output for all covariates eTable 10. Associations between two childhood loneliness items (often feel lonely in childhood; no close friends in childhood) and incident dementia during follow-up eTable 11. Modifying role of adult loneliness in the associations between childhood loneliness and inciden [file jamanetwopen-e2531493-s001.pdf]

# Supplemental Online Content

Wang J, Jiao D, Zhao X, et al. Childhood loneliness and cognitive decline and dementia risk in middle-aged and older adults. *JAMA Netw Open*. 2025;8(9):e2531493. doi:10.1001/jamanetworkopen.2025.31493

**eMethods.**
**eFigure 1.** Flowchart of participant selection
**eFigure 2.** Directed acyclic graph displaying the hypothetical confounding structure of the study
**eFigure 3.** Transition of loneliness from childhood to adulthood
**eFigure 4.** Distribution of global cognition, episodic memory, and executive function scores to assess ceiling effects
**eFigure 5.** Associations of childhood loneliness with rate of cognitive decline and incident dementia across different subgroups
**eTable 1.** Missing data proportions for covariates
**eTable 2.** Baseline characteristics of participants included in the analyses of incident dementia versus those excluded
**eTable 3.** Baseline characteristics of participants included in the analyses of cognitive decline versus those excluded
**eTable 4.** Associations between childhood loneliness and cognitive decline during follow-up in the overall population, with complete regression output for all covariates
**eTable 5.** Associations between two childhood loneliness items (often feel lonely in childhood; no close friends in childhood) and cognitive decline during follow-up
**eTable 6.** Modifying role of adult loneliness in the association between childhood loneliness and cognitive decline during follow-up
**eTable 7.** Associations between childhood loneliness and cognitive decline during follow-up stratified by adult loneliness, with complete regression output for all covariates
**eTable 8.** Mediating role of adult loneliness in the association of childhood loneliness with cognitive decline and incident dementia
**eTable 9.** Associations between childhood loneliness and incident dementia during follow-up in the overall population and stratified by adult loneliness, with complete regression output for all covariates
**eTable 10.** Associations between two childhood loneliness items (often feel lonely in childhood; no close friends in childhood) and incident dementia during follow-up
**eTable 11.** Modifying role of adult loneliness in the associations between childhood loneliness and incident dementia during follow-up
**eTable 12.** Joint associations of childhood and adulthood loneliness with cognitive decline and dementia risk during follow-up
**eTable 13.** Associations between childhood loneliness and cognitive decline during follow-

© 2025 Wang J et al. *JAMA Network Open*.

up after excluding participants with missing data on covariates
**eTable 14.** Associations between childhood loneliness and incident dementia during follow-up after excluding participants with missing data on covariates
**eTable 15.** Associations between childhood loneliness and cognitive decline during follow-up after further adjustment for chronic diseases, depression, and healthy lifestyles
**eTable 16.** Associations between childhood loneliness and incident dementia during follow-up after further adjustment for chronic diseases, depression, and healthy lifestyles
**eTable 17.** Associations between childhood loneliness and cognitive decline during follow-up after excluding participants with cognitive impairment at baseline
**eTable 18.** Associations between childhood loneliness and incident dementia during follow-up after excluding participants with cognitive impairment at baseline
**eTable 19.** Associations between frequency of childhood loneliness and cognitive decline during follow-up
**eTable 20.** Associations between frequency of childhood loneliness and incident dementia during follow-up
**eTable 21.** Associations of childhood loneliness with executive function, assessed using mixed-effects Tobit models to account for ceiling effects
**eReferences.**

This supplemental material has been provided by the authors to give readers additional information about their work.

## eMethods

### Participants selection

Considering the impact of the COVID-19 pandemic on participants' physical and mental health and on survey implementation in the 2020 wave[1-3], which may introduce bias, we restricted our analysis to pre-pandemic waves (2011-2018). A total of 17,707 and 20,542 participants were recruited in the China Health and Retirement Longitudinal Study (CHARLS) 2011 baseline survey and the 2014 early-life survey, respectively, of whom 14,441 participated in both. Among these 14,441 participants, those lacking data on childhood loneliness assessments (n = 538), those with a history of dementia at baseline (n = 240; defined as the coexistence of cognitive impairment and functional impairment or a report by the participant or caregiver of a physician's diagnosis of dementia or related disease), and those lost to all three follow-up assessments between 2013 and 2018 (n = 71) were excluded. A total of 13,592 participants were included in the analyses of the association between childhood loneliness and adult dementia risk.

Of the 13,592 participants, 2,187 lacked baseline cognitive function data and 525 did not complete any of the three follow-up cognitive assessments in 2013, 2015, and 2018. After excluding these participants, 10,880 remained for the analyses of the association between childhood loneliness and adult cognitive decline. Analyses involving adult loneliness were restricted to those with complete adult loneliness data (n = 12,637 for dementia analyses; n = 10,834 for cognitive decline analyses). Missing data for all other covariates were addressed by multiple imputation with chained equations, and thus no additional participants were excluded for covariate missingness.

Baseline characteristics of included and excluded participants are presented in **eTables 2-3 in the Supplement**. Compared with excluded participants, those included were younger and generally healthier, with better cognitive performance, no adult loneliness, and lower prevalence of heart disease, stroke, cancer, and diabetes.

### Assessment of childhood and adult loneliness

Information on early-life experiences before age 17 was collected through face-to-face interviews during the Life History Survey. Based on the Child and Adolescent Psychiatric Assessment (CAPA) and prior research, childhood loneliness was defined as the feeling of being alone and/or friendless[4,5]. Because there is currently no universal diagnostic standard for loneliness, we referenced the dual-item structure used in the UK Biobank questionnaire: "Do you often feel lonely?" and "How often are you able to confide in a close friend?", which has been applied in numerous studies[6-9]. These items were derived from the Revised University of California, Los Angeles (UCLA) Loneliness Scale, the most commonly used multi-item measure of loneliness[6-9], and capture the emotional dimension of loneliness and the perception of intimate relational availability, respectively. Accordingly, we used two analogous key questions based on this structure in CHARLS: (1) "When you were a child, how often did you feel lonely because you had no friends? (often, sometimes, not very often, or never)" and (2) "When you were a child, did you have a close friend? (yes, or no)" Taken together, these items capture both

subjective loneliness and perceived lack of emotional intimacy, thereby reflecting perceived quantitative and qualitative deficits in childhood social relationships.

Responses to each item were converted into binary indicators following UK Biobank research[6-9]. For the loneliness frequency item, a response of "often" was coded as 1 (frequent loneliness), and responses of "sometimes," "not very often," or "never" were coded as 0 (infrequent loneliness). For the close-friendship item, "no" was coded as 1 (absence of a close friend), and "yes" was coded as 0 (presence of a close friend). To increase the specificity of our measure, we combined these binary indicators into a three-category variable: participants were classified as having childhood loneliness only if they reported often feeling lonely and not having a close friend (both items coded 1); those who met only one criterion (only one item coded 1) were classified as having possible childhood loneliness to ensure rigorous categorization; and those reporting neither (both items coded 0) were classified as having no childhood loneliness. Employing both items in the assessment of childhood loneliness may, to some extent, help reduce misclassification. The close-friend item can serve as a complementary indicator reinforcing the loneliness frequency response, which may help improve the recall accuracy of identifying childhood loneliness.

Adult loneliness was assessed using a single item from the 10-item Center for Epidemiological Studies Depression Scale (CES-D-10): 'In the past week, how often did you feel lonely?' Participants were classified as lonely if they reported feeling lonely occasionally (1–2 days/week), frequently (3–4 days/week), or most of the time (5–7 days/week). Those who felt lonely rarely or never (<1 day) were classified as not lonely. This assessment method has been widely used in prior studies[7,10-12]. Notably, individuals without adult loneliness assessments were excluded from the adult loneliness-related analysis.

**Assessment of cognitive function**

Cognitive function was evaluated in two domains: episodic memory and executive function[13,14]. In the episodic memory test, participants were asked to recall 10 unrelated Chinese words immediately (immediate recall) and 5 minutes later (delayed recall) after the examiner read these words. We counted the number of correctly recalled words, and the episodic memory score was the average of the immediate and delayed recall scores, ranging from 0 to 10. In the executive function test, participants were shown a figure and instructed to redraw it. A score of 1 was given for a correct redraw. Participants were then required to subtract 7 from 100 five times consecutively (score ranging, 0–5) and identify the date (year, month, day), season, and day of the week (score ranging, 0–5). The redrawing task score and the scores from these questions were summed to calculate the executive function score, ranging from 0 to 11. The global cognitive score was calculated as the sum of the episodic memory and executive function scores, ranging from 0 to 21. To facilitate direct comparison of cognitive decline across different tests, cognitive scores were standardized to *Z*-scores by subtracting the mean of baseline scores and dividing by the standard deviation (SD) of baseline scores[15]. These *Z*-scores were then used throughout the study as indicators of cognitive function.

**Determination of dementia**

In accordance with prior studies[16-18], dementia was defined using an algorithmic case definition based on the coexistence of cognitive impairment and functional impairment, or a report from the participant

or caregiver of a physician's diagnosis of dementia or related diseases. The definition conforms to Diagnostic and Statistical Manual of Mental Disorders, 5th edition (DSM-5) and the International Classification of Diseases, 10th edition (ICD-10) for dementia diagnosis, and has been validated against clinical diagnoses in a nationwide multisite cross-sectional survey[18]. Participants scoring 1.5 standard deviations below the average cognitive score, adjusted for their education level, were classified as having cognitive impairment[2]. Additionally, based on the Katz-scale, participants who required caregiving assistance with one or more of the six basic activities of daily living (dressing, bathing, cutting food and eating, getting in and out of bed, using the toilet, and controlling urination and defecation) were identified as having functional impairment[16]. If participants did not complete the assessments of cognitive function and functional impairment, a report of a physician's diagnosis of dementia or related diseases was used[16].

In Cox proportional hazards regression models, age was used as the underlying time scale; entry time was defined as each participant's age at baseline, and exit time as age at incident dementia, death, loss to follow-up, or end of follow-up, whichever occurred first[19]. For cases of physician-diagnosed dementia, event age was defined as the age at diagnosis reported by the participant or caregiver. When the exact diagnosis date was unavailable, the age at the corresponding follow-up interview was used. For dementia defined by the concurrent presence of cognitive and functional impairment, event age was defined as the age at the follow-up interview when both criteria were first met.

**Assessment of covariates**

Several potential confounding covariates for adjustment were identified using directed acyclic graphs (DAGs). Several factors were selected based on prior knowledge and existing literature. Specifically, sociodemographic information included age, sex (male and female), and educational attainment (no formal education, junior high school or below, and high school or above). Childhood area of residence was determined by participants' first household registration status and classified as rural or urban[15]. Childhood socioeconomic position was assessed by asking, 'When you were a child before age 17, how did your family's financial situation compare to the average family in your community/village?' Participants were categorized into low (worse than them), medium (same as them), and high (better than them) groups. Health behavior factors included smoking status (non-smoker, former smoker, and current smoker), alcohol consumption (more than once per month, once or less per month, and no consumption), and sleep duration (<7 hours, 7-8 hours, >8 hours). Health status factors included self-reported physician-diagnosed heart diseases (coronary artery disease, heart failure, heart attack, angina, or other heart problems), stroke, cancer, and diabetes. Depressive symptoms were assessed using the 10-item Center for Epidemiological Studies Depression Scale (CES-D-10). Because the CES-D-10 includes an adult-loneliness item that could introduce multicollinearity or overadjustment in subsequent multivariable analyses, we calculated a depression score based on the nine remaining items: bothered by little things; had trouble concentrating; felt depressed; everything was an effort; felt hopeful; felt fearful; sleep was restless; felt happy; and could not get going. Each item was rated on a 0-3 scale, yielding a total depression score ranging from 0 to 27.

As shown in the DAG (**eFigure 2 in the Supplement**), covariates in the minimally sufficient adjustment set included age, sex, educational level, childhood area of residence, and childhood socioeconomic position. Health behavior factors, chronic diseases, and depressive symptoms may serve

as potential mediators. Therefore, we adjusted for these variables only in sensitivity analyses to assess the robustness of the study findings.

**Statistical analysis**

Based on repeated measures of cognitive function, we used linear mixed-effects models to examine the association between childhood loneliness and cognitive decline during follow-up. Regression coefficients ($\beta$) and 95% confidence intervals (95% CI) were calculated, with no childhood loneliness as the reference. The fixed effects in the model included childhood loneliness status, time (follow-up years since baseline), the interaction between childhood loneliness and time, and relevant covariates. Random effects were specified for the intercept and the slope of the time variable at the participant level to account for inter-individual differences in baseline cognitive scores and rates of cognitive change during follow-up. In this framework, the regression coefficients for childhood loneliness reflected differences in baseline cognitive function compared to the reference. The regression coefficient for time represented the annual rate of cognitive change during follow-up in the control group. The regression coefficients for interaction terms reflected differences in the rates of cognitive change during follow-up (additional annual cognitive changes) relative to the reference group.

To examine whether the association between childhood loneliness and cognitive decline rate was modified by adult loneliness, we performed a three-way interaction test[15]. We simultaneously included three two-way interaction terms (childhood loneliness × follow-up time, adult loneliness × follow-up time, and childhood loneliness × adult loneliness) and one three-way interaction term (childhood loneliness × adult loneliness × follow-up time) in our linear mixed-effects models, adjusting for age, sex, educational level, childhood area of residence, and childhood socioeconomic position. A two-way interaction test (childhood loneliness × adult loneliness) in the Cox model was then used to assess the modifying effect of adult loneliness on dementia outcomes. Stratified analyses were further performed by adult loneliness status.

We conducted causal mediation analyses under the counterfactual framework to assess the mediating role of adult loneliness in the associations of childhood loneliness with the rate of cognitive decline and dementia risk. Individual cognitive decline rates were calculated as participant-specific time slopes from linear mixed-effects models with random intercepts and slopes. These continuous rate values were then used as the outcome. The counterfactual framework enabled estimation of the average causal mediation effect (indirect effect, the portion of the association between childhood loneliness and outcomes mediated by adult loneliness), the average direct effect (the portion not mediated by adult loneliness), the total effect (the sum of direct and indirect effects), and the proportion mediated (the proportion of the total effect mediated). Analyses were performed in R using the *CMAverse* package[20]. Effects are presented as regression coefficients for cognitive decline rate and as hazard ratios for dementia outcomes. The term "effects" is used in keeping with mediation-analysis conventions and does not imply a causal interpretation of our findings[21].

We assessed the joint association of childhood and adult loneliness with cognitive decline and dementia risk. We divided participants into six groups based on the transition from childhood to adulthood loneliness status (**eFigure 3 in the Supplement**). Transitions in loneliness status from childhood to adulthood were as follows: 4,460 participants reported neither childhood nor adult

© 2025 Wang J et al. *JAMA Network Open.*

loneliness; 4,219 reported possible childhood loneliness but no adult loneliness; 297 reported childhood loneliness but no adult loneliness; 1,600 reported adult loneliness but no childhood loneliness; 1,835 reported both possible childhood loneliness and adult loneliness; and 226 reported both childhood and adult loneliness.

**Sensitivity analyses**

Multiple sensitivity analyses were conducted to assess the robustness of our findings. First, we reassessed the association after excluding participants with missing covariate data. Second, we further adjusted for chronic diseases (heart diseases, stroke, cancer, and diabetes), depression, and healthy lifestyles (smoking status, alcohol consumption, and sleep duration) to assess whether the associations were independent of these potential mediators. Third, analyses were repeated after excluding participants who still exhibited cognitive impairment at baseline, who are relatively healthier than those with dementia, to further minimize recall bias. Fourth, we examined associations of the original four-level childhood loneliness frequency (never [reference], rarely, sometimes, and often) with each outcome. Fifth, because 15% of participants achieved the highest possible executive function score (**eFigure 4 in the Supplement**)[22], we reassessed the association between childhood loneliness and executive function using mixed-effects Tobit models to account for ceiling effects. Finally, subgroup analyses were conducted based on age, sex, educational level, childhood area of residence, and childhood socioeconomic position to identify other potential moderators.

© 2025 Wang J et al. *JAMA Network Open.*

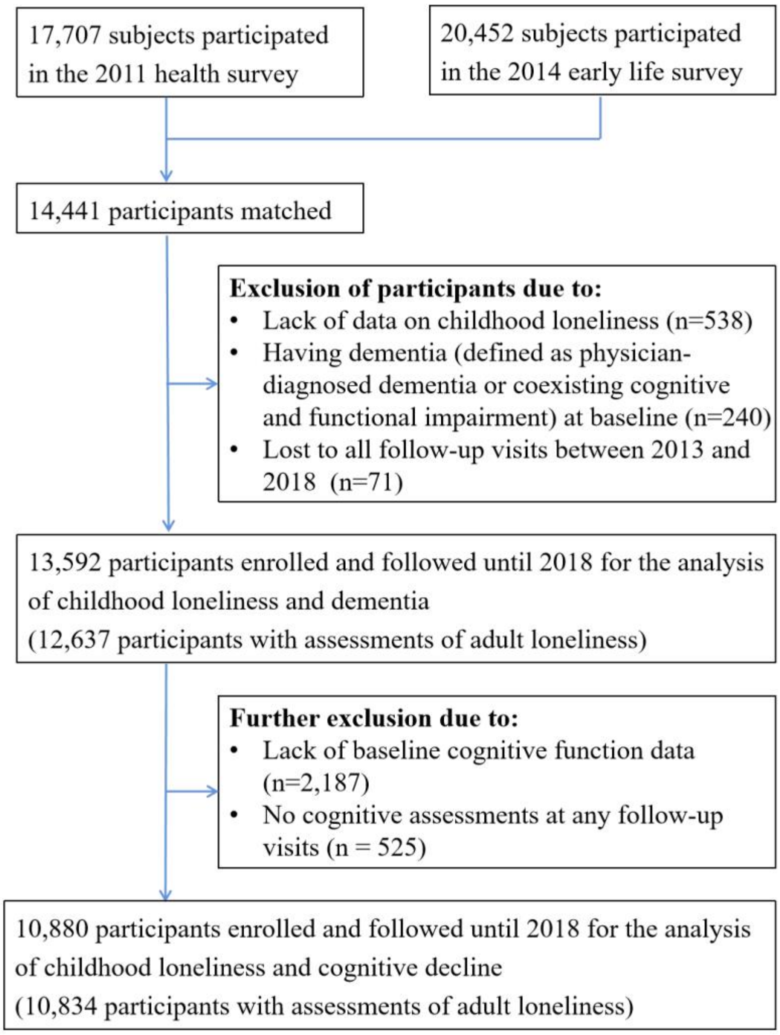


## eFigure 1. Flowchart of participant selection

A total of 13,592 participants were included in the primary analysis of incident dementia, from whom a subset of 10,880 participants with both baseline and follow-up cognitive assessments was selected for the analysis of cognitive decline. Analyses involving adult loneliness were restricted to participants with complete adult loneliness data (n = 12,637 for the dementia analyses; n = 10,834 for the cognitive decline analyses). Missing data for all other covariates were handled by multiple imputation using chained equations, thus no additional participants were excluded for covariate missingness.

© 2025 Wang J et al. *JAMA Network Open*.

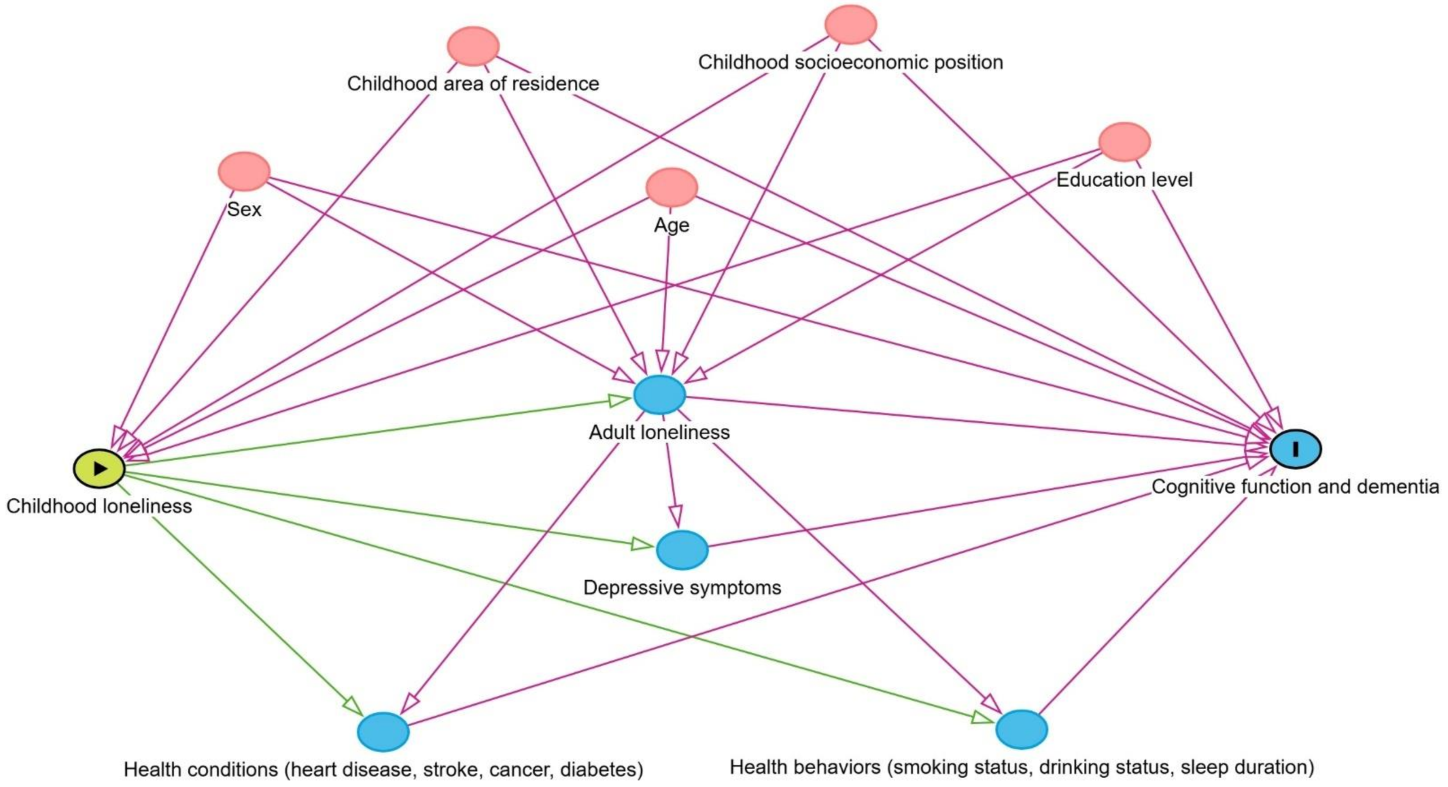


**eFigure 2. Directed Acyclic Graph displaying the hypothetical confounding structure of the study**

© 2025 Wang J et al. *JAMA Network Open*.

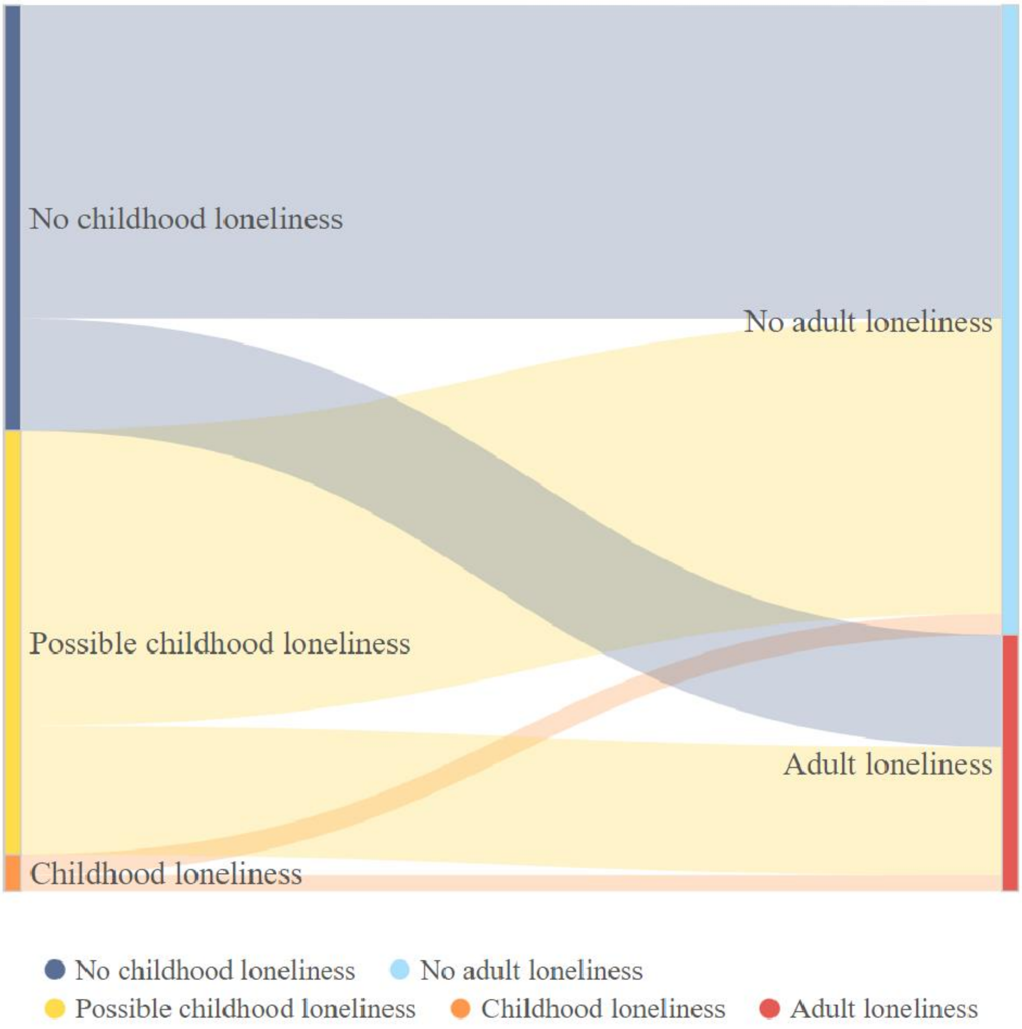


## eFigure 3. Transition of loneliness from childhood to adulthood

This figure illustrates the transition of loneliness status from childhood to adulthood. The transitions are as follows: 4460 participants reported no loneliness in both childhood and adulthood; 4219 participants had possible childhood loneliness but no adult loneliness; 297 participants experienced childhood loneliness but no adult loneliness; 1600 participants had no childhood loneliness but developed adult loneliness; 1835 participants experienced possible childhood loneliness and adult loneliness; and 226 participants reported both childhood and adult loneliness.

© 2025 Wang J et al. *JAMA Network Open*.

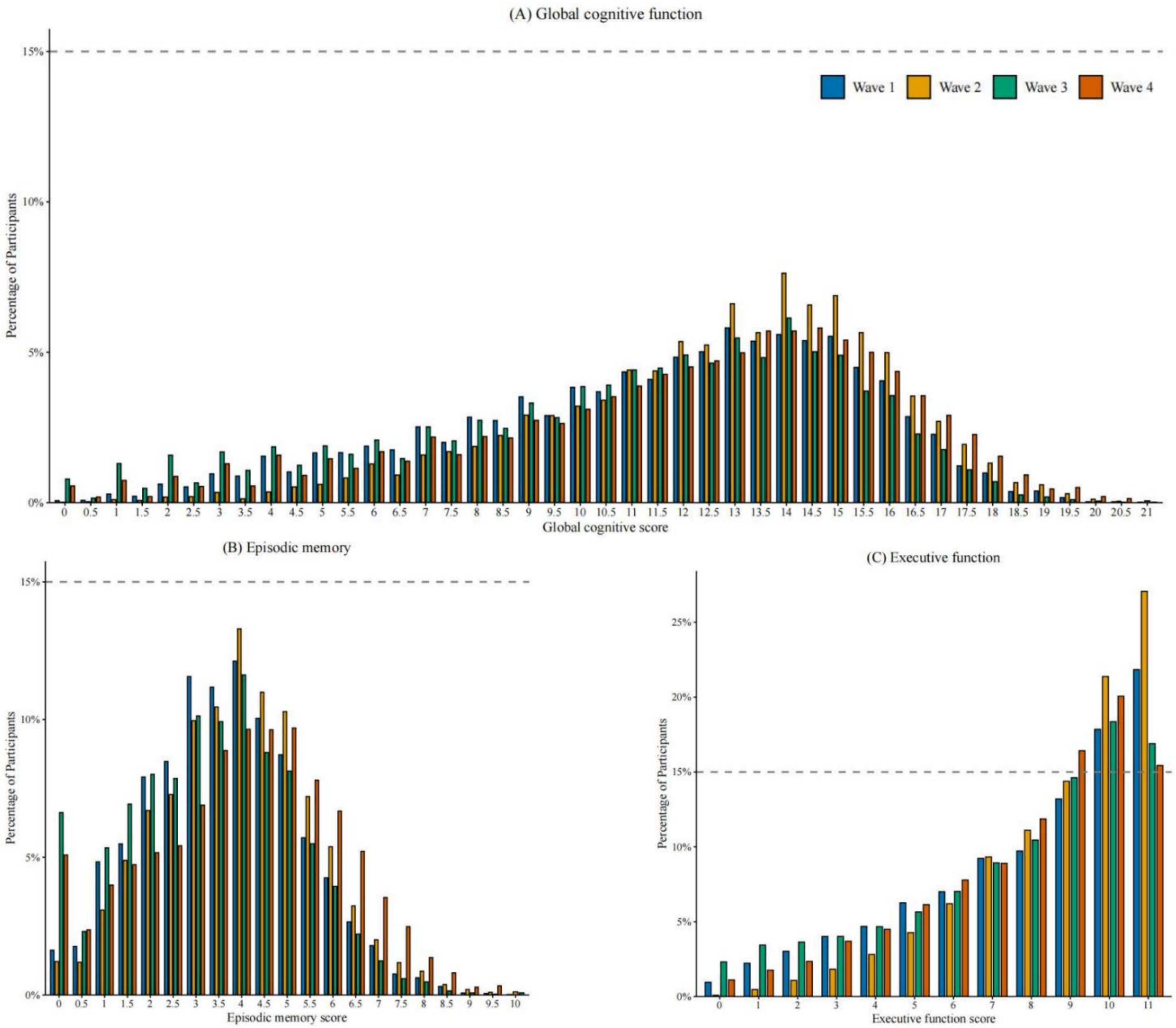


**eFigure 4. Distribution of global cognition, episodic memory, and executive function scores to assess ceiling effects**

Ceiling effects were defined as present when more than 15% of participants achieved the maximum score. Across the four waves, only the executive function test exhibited a ceiling effect.

© 2025 Wang J et al. *JAMA Network Open*.

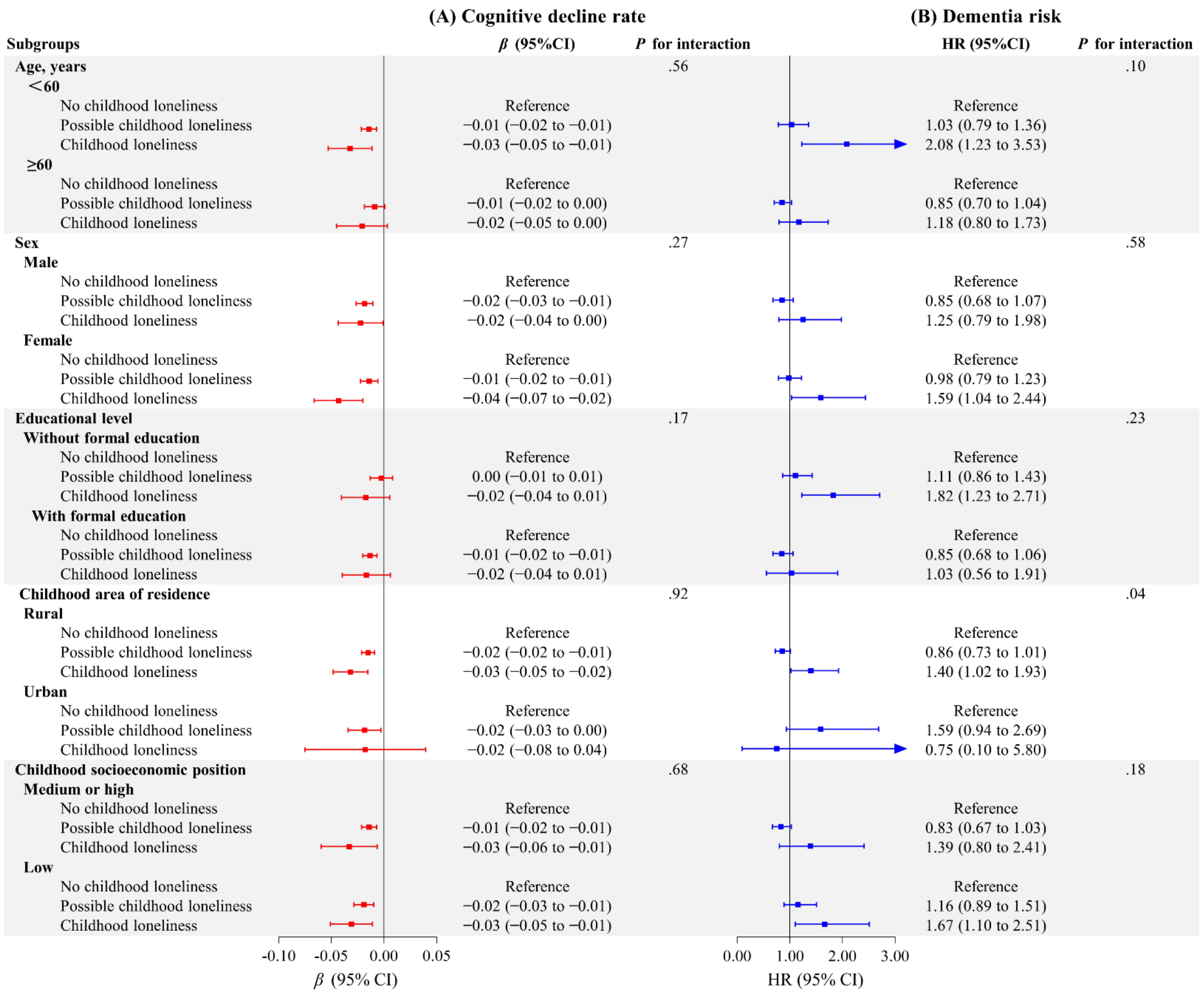


© 2025 Wang J et al. *JAMA Network Open*.

## eFigure 5. Associations of childhood loneliness with rate of cognitive decline and incident dementia across different subgroups

Abbreviations: HR, hazard ratio.

Three-way interaction tests of childhood loneliness × adult loneliness × time were conducted for cognitive decline rate, and two-way interaction tests of childhood loneliness × adult loneliness were conducted for incident dementia, with adjustment for age, sex, educational level, childhood area of residence, and childhood socioeconomic position. Missing data for covariates were handled by multiple imputation with chained equations.

© 2025 Wang J et al. *JAMA Network Open*.

## eTable 1. Missing data proportions for covariates

| Variable | Missing, n (%) [a] |
|---|---|
| Age | 1 (0.0%) |
| Sex | 0 (0.0%) |
| Childhood area of residence | 64 (0.5%) |
| Educational level | 25 (0.2%) |
| Childhood socioeconomic position | 43 (0.3%) |
| Depression score | 56 (0.4%) |
| Smoking status | 69 (0.5%) |
| Drinking status | 72 (0.5%) |
| Sleep duration | 869 (6.4%) |
| Heart-related diseases | 138 (1.0%) |
| Stroke | 97 (0.7%) |
| Cancer | 125 (0.9%) |
| Diabetes | 188 (1.4%) |

[a] Data are presented as No. (%) missing; percentages are based on the total sample (N = 13,592).

© 2025 Wang J et al. *JAMA Network Open*.

**eTable 2. Baseline characteristics of participants included in the analyses of incident dementia versus those excluded [a]**

| Characteristic | Participants, No. (%) | | *P* value [b] |
|---|---|---|---|
| | Excluded (n=4115) | Included (n=13592) | |
| Age, mean (SD), y | 61.43 (12.04) | 58.34 (9.39) | <.001 |
| Sex | | | .001 |
| Male | 2059 (50.1) | 6417 (47.2) | |
| Female | 2053 (49.9) | 7175 (52.8) | |
| Childhood area of residence | | | .02 |
| Rural | 794 (89.0) | 12361 (91.4) | |
| Urban | 98 (11.0) | 1167 (8.6) | |
| Educational level | | | <.001 |
| No formal education | 1785 (43.7) | 6158 (45.4) | |
| Junior high school or below | 1604 (39.3) | 5860 (43.2) | |
| High school or above | 697 (17.1) | 1549 (11.4) | |
| Childhood socioeconomic position | | | .32 |
| High | 73 (9.4) | 1219 (9.0) | |
| Medium | 375 (48.3) | 6925 (51.1) | |
| Low | 328 (42.3) | 5405 (39.9) | |
| Adult loneliness status | | | .001 |
| No adult loneliness | 2233 (68.0) | 8976 (71.0) | |
| Adult loneliness | 1052 (32.0) | 3661 (29.0) | |
| Depression score, mean (SD) | 6.60 (6.29) | 7.34 (5.87) | <.001 |
| Smoking status | | | .17 |
| Current smoker | 1255 (31.1) | 4259 (31.5) | |
| Former smoker | 360 (8.9) | 1080 (8.0) | |
| Non-smoker | 2421 (60.0) | 8184 (60.5) | |
| Drinking status | | | .003 |
| > once a month | 925 (22.9) | 3458 (25.6) | |
| ≤ once a month | 331 (8.2) | 1053 (7.8) | |
| None of these | 2776 (68.8) | 9009 (66.6) | |
| Sleep duration | | | .01 |
| <7 hours | 1721 (51.1) | 6384 (50.2) | |
| 7–8 hours | 1328 (39.4) | 5297 (41.6) | |
| >8 hours | 319 (9.5) | 1042 (8.2) | |
| Chronic diseases | | | |
| Heart-related diseases | 568 (14.2) | 1525 (11.3) | <.001 |
| Stroke | 190 (4.7) | 223 (1.7) | <.001 |
| Cancer | 55 (1.4) | 125 (0.9) | .02 |
| Diabetes | 277 (6.9) | 721 (5.4) | <.001 |
| Global cognitive z-scores, mean (SD) | -0.07 (1.12) | 0.02 (0.97) | <.001 |
| Episodic memory z-scores, mean (SD) | -0.06 (1.09) | 0.02 (0.98) | <.001 |
| Executive function z-scores, mean (SD) | -0.06 (1.08) | 0.01 (0.98) | <.001 |

© 2025 Wang J et al. *JAMA Network Open.*

Abbreviations: SD, standard deviation.

[a] Percentages may not total 100% because of rounding. Counts may not sum to column totals because of missing data.

[b] Continuous variables were compared using one-way ANOVA test or Kruskal-Wallis test. Categorical variables were compared using $\chi^2$ test or Fisher's exact test.

**eTable 3. Baseline characteristics of participants included in the analyses of cognitive decline versus those excluded [a]**

| Characteristic | Participants, No. (%) | | *P* value [b] |
|---|---|---|---|
| | Excluded (n=6827) | Included (n=10880) | |
| Age, mean (SD), y | 60.88 (11.59) | 57.91 (8.95) | <.001 |
| Sex | | | .19 |
| Male | 3310 (48.5) | 5166 (47.5) | |
| Female | 3514 (51.5) | 5714 (52.5) | |
| Childhood area of residence | | | <.001 |
| Rural | 3327 (92.8) | 9828 (90.7) | |
| Urban | 260 (7.2) | 1005 (9.3) | |
| Educational level | | | <.001 |
| No formal education | 3299 (48.7) | 4644 (42.7) | |
| Junior high school or below | 2552 (37.7) | 4912 (45.2) | |
| High school or above | 924 (13.6) | 1322 (12.2) | |
| Childhood socioeconomic position | | | .003 |
| High | 326 (9.4) | 966 (8.9) | |
| Medium | 1681 (48.4) | 5619 (51.8) | |
| Low | 1463 (42.2) | 4270 (39.3) | |
| Adult loneliness status | | | <.001 |
| No adult loneliness | 3369 (66.2) | 7840 (72.4) | |
| Adult loneliness | 1719 (33.8) | 2994 (27.6) | |
| Depression score, mean (SD) | 6.38 (6.30) | 7.65 (5.72) | <.001 |
| Smoking status | | | .01 |
| Current smoker | 2162 (32.4) | 3352 (30.8) | |
| Former smoker | 504 (7.5) | 936 (8.6) | |
| Non-smoker | 4014 (60.1) | 6591 (60.6) | |
| Drinking status | | | .001 |
| > once a month | 1561 (23.4) | 2822 (25.9) | |
| ≤ once a month | 534 (8.0) | 850 (7.8) | |
| None of these | 4577 (68.6) | 7208 (66.2) | |
| Sleep duration | | | <.001 |
| <7 hours | 2728 (51.8) | 5377 (49.7) | |
| 7–8 hours | 2055 (39.0) | 4570 (42.2) | |
| >8 hours | 486 (9.2) | 875 (8.1) | |
| Chronic diseases | | | |
| Heart-related diseases | 832 (12.5) | 1261 (11.6) | .08 |
| Stroke | 236 (3.5) | 177 (1.6) | <.001 |
| Cancer | 79 (1.2) | 101 (0.9) | .12 |
| Diabetes | 399 (6.0) | 599 (5.6) | .20 |
| Global cognitive z-scores, mean (SD) | -0.18 (1.14) | 0.06 (0.95) | <.001 |
| Episodic memory z-scores, mean (SD) | -0.14 (1.09) | 0.04 (0.97) | <.001 |
| Executive function z-scores, mean (SD) | -0.17 (1.11) | 0.05 (0.96) | <.001 |

© 2025 Wang J et al. *JAMA Network Open*.

Abbreviations: SD, standard deviation.

[a] Percentages may not total 100% because of rounding. Counts may not sum to column totals because of missing data.

[b] Continuous variables were compared using one-way ANOVA test or Kruskal-Wallis test. Categorical variables were compared using $\chi^2$ test or Fisher's exact test.

© 2025 Wang J et al. *JAMA Network Open*.

**eTable 4. Associations between childhood loneliness and cognitive decline during follow-up in the overall population, with complete regression output for all covariates**

| Variable | Global cognitive function | | Episodic memory | | Executive function | |
|---|---|---|---|---|---|---|
| | *β* (95% CI) [a] | *P* | *β* (95% CI) [a] | *P* | *β* (95% CI) [a] | *P* |
| **Model 1** | | | | | | |
| Childhood loneliness status [b] | | | | | | |
| No childhood loneliness | 0 [Reference] | NA | 0 [Reference] | NA | 0 [Reference] | NA |
| Possible childhood loneliness | -0.08 (-0.11 to -0.05) | <.001 | -0.05 (-0.09 to -0.02) | .002 | -0.07 (-0.10 to -0.04) | <.001 |
| Childhood loneliness | -0.14 (-0.21 to -0.06) | <.001 | 0.03 (-0.06 to 0.11) | .52 | -0.19 (-0.27 to -0.11) | <.001 |
| Time [c] | -0.03 (-0.03 to -0.02) | <.001 | 0.00 (0.00 to 0.01) | .52 | -0.04 (-0.04 to -0.03) | <.001 |
| Childhood loneliness status × time [c] | | | | | | |
| No childhood loneliness | 0 [Reference] | NA | 0 [Reference] | NA | 0 [Reference] | NA |
| Possible childhood loneliness | -0.02 (-0.02 to -0.01) | <.001 | -0.02 (-0.03 to -0.02) | <.001 | -0.01 (-0.01 to 0.00) | .005 |
| Childhood loneliness | -0.03 (-0.05 to -0.02) | <.001 | -0.05 (-0.07 to -0.03) | <.001 | -0.01 (-0.03 to 0.00) | .06 |
| Age [c] | -0.02 (-0.02 to -0.02) | <.001 | -0.03 (-0.03 to -0.02) | <.001 | -0.01 (-0.01 to -0.01) | <.001 |
| Sex [b] | | | | | | |
| Male | 0 [Reference] | NA | 0 [Reference] | NA | 0 [Reference] | NA |
| Female | -0.21 (-0.24 to -0.18) | <.001 | 0.07 (0.04 to 0.10) | <.001 | -0.32 (-0.34 to -0.29) | <.001 |
| Childhood area of residence [b] | | | | | | |
| Urban | 0 [Reference] | NA | 0 [Reference] | NA | 0 [Reference] | NA |
| Rural | -0.33 (-0.38 to -0.29) | <.001 | -0.32 (-0.36 to -0.27) | <.001 | -0.27 (-0.31 to -0.22) | <.001 |
| Educational level [b] | | | | | | |
| No formal education | 0 [Reference] | NA | 0 [Reference] | NA | 0 [Reference] | NA |
| Junior high school or below | 0.84 (0.81 to 0.87) | <.001 | 0.52 (0.48 to 0.55) | <.001 | 0.83 (0.79 to 0.86) | <.001 |
| High school or above | 1.19 (1.14 to 1.24) | <.001 | 0.91 (0.86 to 0.96) | <.001 | 1.07 (1.02 to 1.12) | <.001 |
| Childhood socioeconomic position [b] | | | | | | |
| High | 0 [Reference] | NA | 0 [Reference] | NA | 0 [Reference] | NA |
| Medium | -0.02 (-0.07 to 0.03) | .46 | -0.05 (-0.10 to 0.00) | .04 | 0.00 (-0.05 to 0.05) | .95 |
| Low | -0.08 (-0.13 to -0.03) | .001 | -0.08 (-0.13 to -0.03) | .002 | -0.07 (-0.12 to -0.02) | .008 |

© 2025 Wang J et al. *JAMA Network Open*.

| | | | | | | |
|---|---|---|---|---|---|---|
| **Model 2** | | | | | | |
| Childhood loneliness status [b] | | | | | | |
| No childhood loneliness | 0 [Reference] | NA | 0 [Reference] | NA | 0 [Reference] | NA |
| Possible childhood loneliness | -0.07 (-0.11 to -0.04) | <.001 | -0.05 (-0.08 to -0.02) | .003 | -0.07 (-0.10 to -0.04) | <.001 |
| Childhood loneliness | -0.11 (-0.19 to -0.03) | .006 | 0.05 (-0.04 to 0.13) | .31 | -0.16 (-0.24 to -0.09) | <.001 |
| Time [c] | -0.03 (-0.03 to -0.02) | <.001 | 0.00 (0.00 to 0.01) | .50 | -0.04 (-0.04 to -0.03) | <.001 |
| Childhood loneliness status × time [c] | | | | | | |
| No childhood loneliness | 0 [Reference] | NA | 0 [Reference] | NA | 0 [Reference] | NA |
| Possible childhood loneliness | -0.02 (-0.02 to -0.01) | <.001 | -0.02 (-0.03 to -0.02) | <.001 | -0.01 (-0.01 to 0.00) | .004 |
| Childhood loneliness | -0.03 (-0.05 to -0.02) | <.001 | -0.05 (-0.07 to -0.03) | <.001 | -0.01 (-0.03 to 0.00) | .06 |
| Age [c] | -0.02 (-0.02 to -0.02) | <.001 | -0.03 (-0.03 to -0.02) | <.001 | -0.01 (-0.01 to -0.01) | <.001 |
| Sex [b] | | | | | | |
| Male | 0 [Reference] | NA | 0 [Reference] | NA | 0 [Reference] | NA |
| Female | -0.20 (-0.22 to -0.17) | <.001 | 0.08 (0.05 to 0.11) | <.001 | -0.30 (-0.33 to -0.27) | <.001 |
| Childhood area of residence [b] | | | | | | |
| Urban | 0 [Reference] | NA | 0 [Reference] | NA | 0 [Reference] | NA |
| Rural | -0.33 (-0.37 to -0.28) | <.001 | -0.31 (-0.36 to -0.26) | <.001 | -0.26 (-0.30 to -0.21) | <.001 |
| Educational level [b] | | | | | | |
| No formal education | 0 [Reference] | NA | 0 [Reference] | NA | 0 [Reference] | NA |
| Junior high school or below | 0.82 (0.79 to 0.85) | <.001 | 0.50 (0.47 to 0.53) | <.001 | 0.81 (0.78 to 0.84) | <.001 |
| High school or above | 1.17 (1.12 to 1.21) | <.001 | 0.89 (0.85 to 0.94) | <.001 | 1.05 (1.00 to 1.10) | <.001 |
| Childhood socioeconomic position [b] | | | | | | |
| High | 0 [Reference] | NA | 0 [Reference] | NA | 0 [Reference] | NA |
| Medium | -0.02 (-0.06 to 0.03) | .54 | -0.05 (-0.10 to 0.00) | .05 | 0.00 (-0.04 to 0.05) | .85 |
| Low | -0.07 (-0.12 to -0.02) | .004 | -0.07 (-0.12 to -0.02) | .004 | -0.06 (-0.11 to -0.01) | .02 |
| Adult loneliness [b] | | | | | | |
| No adult loneliness | 0 [Reference] | NA | 0 [Reference] | NA | 0 [Reference] | NA |
| Adult loneliness | -0.19 (-0.22 to -0.16) | <.001 | -0.12 (-0.15 to -0.09) | <.001 | -0.18 (-0.21 to -0.15) | <.001 |

Abbreviations: NA, not applicable.

[a] The $\beta$ coefficients were estimated using linear mixed-effects models. Model 1 was adjusted for age, sex, educational level, childhood area of residence, and childhood socioeconomic position. Model 2 was

© 2025 Wang J et al. *JAMA Network Open*.

further adjusted for adult loneliness, which was included only among the 10,834 participants with available adult loneliness data. Missing data for other covariates were handled by multiple imputation with chained equations.

[b] The $\beta$ coefficient and its 95% CI are reported as SD.

[c] The $\beta$ coefficient and its 95% CI are reported as SD per year.

© 2025 Wang J et al. *JAMA Network Open*.

**eTable 5. Associations between two childhood loneliness items (often feel lonely in childhood; no close friends in childhood) and cognitive decline during follow-up**

| Variable | Global cognitive function | | Episodic memory | | Executive function | |
|---|---|---|---|---|---|---|
| | *β* (95% CI)[a] | *P* | *β* (95% CI)[a] | *P* | *β* (95% CI)[a] | *P* |
| **Model 1** | | | | | | |
| Often feel lonely in childhood | | | | | | |
| Item[b] | -0.09 (-0.15 to -0.02) | .006 | 0.04 (-0.03 to 0.10) | .30 | -0.13 (-0.19 to -0.07) | <.001 |
| Item × time[c] | -0.02 (-0.03 to -0.01) | .003 | -0.03 (-0.05 to -0.01) | <.001 | -0.01 (-0.02 to 0.00) | .16 |
| No close friends in childhood | | | | | | |
| Item[b] | -0.07 (-0.10 to -0.04) | <.001 | -0.05 (-0.08 to -0.01) | .004 | -0.06 (-0.09 to -0.03) | <.001 |
| Item × time[c] | -0.02 (-0.02 to -0.01) | <.001 | -0.02 (-0.03 to -0.02) | <.001 | -0.01 (-0.01 to 0.00) | .007 |
| Time[c] | -0.03 (-0.03 to -0.02) | <.001 | 0.00 (0.00 to 0.01) | .51 | -0.04 (-0.04 to -0.03) | <.001 |
| **Model 2** | | | | | | |
| Often feel lonely in childhood | | | | | | |
| Item[b] | -0.06 (-0.12 to 0.00) | .05 | 0.05 (-0.02 to 0.12) | .13 | -0.11 (-0.17 to -0.04) | <.001 |
| Item × time[c] | -0.02 (-0.03 to -0.01) | .005 | -0.03 (-0.05 to -0.01) | <.001 | -0.01 (-0.02 to 0.00) | .16 |
| No close friends in childhood | | | | | | |
| Item[b] | -0.07 (-0.10 to -0.04) | <.001 | -0.05 (-0.08 to -0.01) | .006 | -0.06 (-0.09 to -0.03) | <.001 |
| Item × time[c] | -0.02 (-0.02 to -0.01) | <.001 | -0.02 (-0.03 to -0.02) | <.001 | -0.01 (-0.01 to 0.00) | .005 |
| Time[c] | -0.03 (-0.03 to -0.02) | <.001 | 0.00 (0.00 to 0.01) | .51 | -0.04 (-0.04 to -0.03) | <.001 |

[a] The *β* coefficients were estimated using linear mixed-effects models. Model 1 was adjusted for age, sex, educational level, childhood area of residence, and childhood socioeconomic position. Model 2 was further adjusted for adult loneliness, which was included only among the 10,834 participants with available adult loneliness data. Missing data for other covariates were handled by multiple imputation with chained equations. Models for each childhood-loneliness item were adjusted for the other item and its interaction with time.

[b] The *β* coefficient and its 95% CI are reported as SD.

[c] The *β* coefficient and its 95% CI are reported as SD per year.

© 2025 Wang J et al. *JAMA Network Open*.

**eTable 6. Modifying role of adult loneliness in the association between childhood loneliness and cognitive decline during follow-up**

| Covariate | Global cognitive function | | Episodic memory | | Executive function | |
|---|---|---|---|---|---|---|
| | *β* (95% CI) [a] | *P* | *β* (95% CI) [a] | *P* | *β* (95% CI) [a] | *P* |
| Childhood loneliness status [b] | | | | | | |
| No childhood loneliness | 0 [Reference] | NA | 0 [Reference] | NA | 0 [Reference] | NA |
| Possible childhood loneliness | -0.06 (-0.10 to -0.03) | <.001 | -0.05 (-0.08 to -0.01) | .02 | -0.05 (-0.09 to -0.02) | .003 |
| Childhood loneliness | -0.11 (-0.21 to -0.01) | .03 | 0.03 (-0.08 to 0.14) | .60 | -0.16 (-0.26 to -0.06) | .002 |
| Adult loneliness [b] | -0.17 (-0.22 to -0.12) | <.001 | -0.09 (-0.15 to -0.04) | <.001 | -0.17 (-0.22 to -0.13) | <.001 |
| Time [c] | -0.03 (-0.03 to -0.02) | <.001 | 0.00 (0.00 to 0.01) | .26 | -0.04 (-0.04 to -0.03) | <.001 |
| Childhood loneliness status × time [c] | | | | | | |
| No childhood loneliness | 0 [Reference] | NA | 0 [Reference] | NA | 0 [Reference] | NA |
| Possible childhood loneliness | -0.02 (-0.02 to -0.01) | <.001 | -0.02 (-0.03 to -0.01) | <.001 | -0.01 (-0.02 to 0.00) | .004 |
| Childhood loneliness | -0.04 (-0.06 to -0.02) | <.001 | -0.05 (-0.08 to -0.03) | <.001 | -0.02 (-0.03 to 0.00) | .10 |
| Adult loneliness× time [c] | 0.00 (-0.01 to 0.01) | .86 | -0.01 (-0.02 to 0.01) | .27 | 0.01 (0.00 to 0.01) | .21 |
| Childhood loneliness status × adult loneliness [b] | | | | | | |
| No childhood loneliness | 0 [Reference] | NA | 0 [Reference] | NA | 0 [Reference] | NA |
| Possible childhood loneliness | -0.05 (-0.11 to 0.02) | .16 | -0.02 (-0.10 to 0.05) | .56 | -0.05 (-0.12 to 0.02) | .13 |
| Childhood loneliness | 0.00 (-0.16 to 0.16) | >.99 | 0.02 (-0.15 to 0.20) | .79 | -0.01 (-0.17 to 0.14) | .86 |
| Childhood loneliness status × adult loneliness× time [c] | | | | | | |
| No childhood loneliness | 0 [Reference] | NA | 0 [Reference] | NA | 0 [Reference] | NA |
| Possible childhood loneliness | 0.00 (-0.01 to 0.02) | .72 | 0.00 (-0.02 to 0.02) | .99 | 0.00 (-0.01 to 0.02) | .59 |
| Childhood loneliness | 0.01 (-0.02 to 0.04) | .62 | 0.01 (-0.03 to 0.05) | .68 | 0.00 (-0.03 to 0.03) | .87 |
| Age [c] | -0.02 (-0.02 to -0.02) | <.001 | -0.03 (-0.03 to -0.02) | <.001 | -0.01 (-0.01 to -0.01) | <.001 |
| Sex [b] | | | | | | |
| Male | 0 [Reference] | NA | 0 [Reference] | NA | 0 [Reference] | NA |
| Female | -0.20 (-0.22 to -0.17) | <.001 | 0.08 (0.05 to 0.11) | <.001 | -0.30 (-0.33 to -0.28) | <.001 |
| Childhood area of residence [b] | | | | | | |

© 2025 Wang J et al. *JAMA Network Open*.

| | | | | | | |
|---|---|---|---|---|---|---|
| Urban | 0 [Reference] | NA | 0 [Reference] | NA | 0 [Reference] | NA |
| Rural | -0.33 (-0.37 to -0.28) | <.001 | -0.31 (-0.36 to -0.26) | <.001 | -0.26 (-0.31 to -0.21) | <.001 |
| Educational level [b] | | | | | | |
| No formal education | 0 [Reference] | NA | 0 [Reference] | NA | 0 [Reference] | NA |
| Junior high school or below | 0.82 (0.79 to 0.85) | <.001 | 0.50 (0.47 to 0.53) | <.001 | 0.81 (0.78 to 0.84) | <.001 |
| High school or above | 1.17 (1.12 to 1.21) | <.001 | 0.89 (0.85 to 0.94) | <.001 | 1.05 (1.00 to 1.10) | <.001 |
| Childhood socioeconomic position [b] | | | | | | |
| High | 0 [Reference] | NA | 0 [Reference] | NA | 0 [Reference] | NA |
| Medium | -0.01 (-0.06 to 0.03) | .57 | -0.05 (-0.10 to 0.00) | .05 | 0.01 (-0.04 to 0.05) | .82 |
| Low | -0.07 (-0.12 to -0.02) | .004 | -0.07 (-0.12 to -0.02) | .004 | -0.06 (-0.11 to -0.01) | .02 |

Abbreviations: NA, not applicable.

[a] The *β* coefficient was estimated using linear mixed-effects models adjusted for age, sex, educational level, childhood area of residence, and childhood socioeconomic position. A total of 10,834 participants with assessments of adult loneliness were included in adjusted models. Missing values for other covariates were handled by multiple imputation with chained equations.

[b] The *β* coefficient and its 95% CI are reported as SD.

[c] The *β* coefficient and its 95% CI are reported as SD per year.

© 2025 Wang J et al. *JAMA Network Open*.

**eTable 7. Associations between childhood loneliness and cognitive decline during follow-up stratified by adult loneliness, with complete regression output for all covariates**

| Variable | Global cognitive function | | Episodic memory | | Executive function | |
|---|---|---|---|---|---|---|
| | **β (95% CI) [a]** | ***P*** | **β (95% CI) [a]** | ***P*** | **β (95% CI) [a]** | ***P*** |
| **Adult non-loneliness group (n=7840)** | | | | | | |
| Childhood loneliness status [b] | | | | | | |
| No childhood loneliness | 0 [Reference] | NA | 0 [Reference] | NA | 0 [Reference] | NA |
| Possible childhood loneliness | -0.07 (-0.11 to -0.04) | <.001 | -0.05 (-0.09 to -0.01) | .02 | -0.06 (-0.10 to -0.03) | <.001 |
| Childhood loneliness | -0.13 (-0.23 to -0.02) | .02 | 0.03 (-0.09 to 0.14) | .63 | -0.18 (-0.28 to -0.08) | <.001 |
| Time [c] | -0.03 (-0.03 to -0.02) | <.001 | 0.00 (0.00 to 0.01) | .25 | -0.04 (-0.04 to -0.03) | <.001 |
| Childhood loneliness status × time [c] | | | | | | |
| No childhood loneliness | 0 [Reference] | NA | 0 [Reference] | NA | 0 [Reference] | NA |
| Possible childhood loneliness | -0.02 (-0.02 to -0.01) | <.001 | -0.02 (-0.03 to -0.01) | <.001 | -0.01 (-0.02 to 0.00) | .004 |
| Childhood loneliness | -0.04 (-0.06 to -0.02) | <.001 | -0.05 (-0.08 to -0.03) | <.001 | -0.02 (-0.03 to 0.00) | .10 |
| Age [c] | -0.02 (-0.02 to -0.01) | <.001 | -0.02 (-0.03 to -0.02) | <.001 | -0.01 (-0.01 to -0.01) | <.001 |
| Sex [b] | | | | | | |
| Male | 0 [Reference] | NA | 0 [Reference] | NA | 0 [Reference] | NA |
| Female | -0.18 (-0.21 to -0.15) | <.001 | 0.09 (0.05 to 0.12) | <.001 | -0.29 (-0.32 to -0.26) | <.001 |
| Childhood area of residence [b] | | | | | | |
| Urban | 0 [Reference] | NA | 0 [Reference] | NA | 0 [Reference] | NA |
| Rural | -0.31 (-0.36 to -0.25) | <.001 | -0.30 (-0.35 to -0.25) | <.001 | -0.24 (-0.29 to -0.19) | <.001 |
| Educational level [b] | | | | | | |
| No formal education | 0 [Reference] | NA | 0 [Reference] | NA | 0 [Reference] | NA |
| Junior high school or below | 0.80 (0.76 to 0.83) | <.001 | 0.52 (0.48 to 0.56) | <.001 | 0.77 (0.74 to 0.81) | <.001 |
| High school or above | 1.15 (1.09 to 1.20) | <.001 | 0.90 (0.85 to 0.96) | <.001 | 1.02 (0.96 to 1.07) | <.001 |
| Childhood socioeconomic position [b] | | | | | | |
| High | 0 [Reference] | NA | 0 [Reference] | NA | 0 [Reference] | NA |
| Medium | 0.00 (-0.06 to 0.05) | .90 | -0.03 (-0.08 to 0.03) | .32 | 0.01 (-0.04 to 0.06) | .74 |
| Low | -0.07 (-0.13 to -0.02) | .01 | -0.06 (-0.12 to 0.00) | .04 | -0.06 (-0.12 to -0.01) | .02 |
| **Adult loneliness group (n=2994)** | | | | | | |

© 2025 Wang J et al. *JAMA Network Open*.

| | | | | | | |
|---|---|---|---|---|---|---|
| Childhood loneliness status [b] | | | | | | |
| No childhood loneliness | 0 [Reference] | NA | 0 [Reference] | NA | 0 [Reference] | NA |
| Possible childhood loneliness | -0.09 (-0.15 to -0.03) | .003 | -0.06 (-0.12 to 0.00) | 0.06 | -0.08 (-0.14 to -0.02) | .01 |
| Childhood loneliness | -0.09 (-0.22 to 0.04) | .16 | 0.05 (-0.08 to 0.18) | 0.44 | -0.14 (-0.27 to -0.01) | .03 |
| Time [c] | -0.03 (-0.03 to -0.02) | <.001 | 0.00 (-0.01 to 0.01) | 0.49 | -0.03 (-0.04 to -0.02) | <.001 |
| Childhood loneliness status × time [c] | | | | | | |
| No childhood loneliness | 0 [Reference] | NA | 0 [Reference] | NA | 0 [Reference] | NA |
| Possible childhood loneliness | -0.01 (-0.03 to 0.00) | .01 | -0.02 (-0.04 to -0.01) | .002 | -0.01 (-0.02 to 0.01) | .31 |
| Childhood loneliness | -0.03 (-0.05 to 0.00) | .04 | -0.04 (-0.08 to -0.01) | .01 | -0.01 (-0.04 to 0.01) | .28 |
| Age [c] | -0.02 (-0.02 to -0.02) | <.001 | -0.03 (-0.03 to -0.03) | <.001 | -0.01 (-0.02 to -0.01) | <.001 |
| Sex [b] | | | | | | |
| Male | 0 [Reference] | NA | 0 [Reference] | NA | 0 [Reference] | NA |
| Female | -0.23 (-0.29 to -0.18) | <.001 | 0.06 (0.01 to 0.12) | .02 | -0.34 (-0.40 to -0.29) | <.001 |
| Childhood area of residence [b] | | | | | | |
| Urban | 0 [Reference] | NA | 0 [Reference] | NA | 0 [Reference] | NA |
| Rural | -0.39 (-0.49 to -0.28) | <.001 | -0.34 (-0.44 to -0.24) | <.001 | -0.32 (-0.43 to -0.21) | <.001 |
| Educational level [b] | | | | | | |
| No formal education | 0 [Reference] | NA | 0 [Reference] | NA | 0 [Reference] | NA |
| Junior high school or below | 0.87 (0.81 to 0.92) | <.001 | 0.46 (0.40 to 0.52) | <.001 | 0.90 (0.84 to 0.96) | <.001 |
| High school or above | 1.23 (1.13 to 1.34) | <.001 | 0.89 (0.79 to 0.99) | <.001 | 1.15 (1.04 to 1.25) | <.001 |
| Childhood socioeconomic position [b] | | | | | | |
| High | 0 [Reference] | NA | 0 [Reference] | NA | 0 [Reference] | NA |
| Medium | -0.04 (-0.14 to 0.06) | .38 | -0.10 (-0.20 to -0.01) | .03 | -0.01 (-0.11 to 0.10) | .92 |
| Low | -0.08 (-0.18 to 0.02) | .14 | -0.11 (-0.21 to -0.01) | .03 | -0.04 (-0.14 to 0.06) | .44 |

Abbreviations: NA, not applicable.

[a] The $\beta$ coefficients were estimated using linear mixed-effects models adjusted for age, sex, educational level, childhood area of residence, and childhood socioeconomic position. Missing covariate data were handled by multiple imputation with chained equations.

[b] The $\beta$ coefficient and its 95% CI are reported as SD.

[c] The $\beta$ coefficient and its 95% CI are reported as SD per year.

© 2025 Wang J et al. *JAMA Network Open*.

## eTable 8. Mediating role of adult loneliness in the association of childhood loneliness with cognitive decline and incident dementia

| Exposure | Cognitive decline rate [a] | | Incident dementia | |
|---|---|---|---|---|
| | *β* (95% CI) [b] | *P* | HR (95% CI) [b] | *P* |
| **Possible childhood loneliness** | | | | |
| Total effect | -0.0039 (-0.0051 to -0.0027) | <.001 | 0.96 (0.82 to 1.14) | .67 |
| ACME [c] | -0.0001 (-0.0002 to 0.0000) | .02 | 1.01 (1.00 to 1.02) | .04 |
| ADE [d] | -0.0038 (-0.0050 to -0.0026) | <.001 | 0.96 (0.81 to 1.13) | .59 |
| Proportion mediated, % [e] | 2.3 (0.2 to 4.4) | .03 | –27.1 (–158.6 to 104.5) | .69 |
| **Childhood loneliness** | | | | |
| Total effect | -0.0067 (-0.0099 to -0.0035) | <.001 | 1.57 (1.14 to 2.16) | .006 |
| ACME [c] | -0.0006 (-0.0008 to -0.0003) | <.001 | 1.07 (1.03 to 1.10) | <.001 |
| ADE [d] | -0.0062 (-0.0093 to -0.0030) | <.001 | 1.47 (1.07 to 2.03) | .02 |
| Proportion mediated, % [e] | 8.5 (2.9 to 14.1） | .003 | 17.2 (4.9 to 29.5) | .006 |

Abbreviations: HR, hazard ratio; ACME, average causal mediation effect; ADE, average direct effect.

[a] Individual cognitive decline rates were calculated as participant-specific time slopes from a linear mixed-effects model with random intercepts and slopes.

[b] The *β* coefficients (95% CIs) were estimated using linear regression models, and HRs (95% CIs) were estimated using Cox proportional hazards regression models with follow-up time as the timescale. Models were adjusted for age, sex, educational level, childhood area of residence, and childhood socioeconomic position. Mediation analyses were restricted to participants with complete adult loneliness data (n = 10,834 for cognitive decline; n = 12,637 for incident dementia). Missing data for covariates were handled by multiple imputation with chained equations.

[c] The ACME represents the indirect effect of childhood loneliness on the outcome mediated through adult loneliness.

[d] The ADE represents the direct effect of childhood loneliness on the outcome not mediated by adult loneliness.

[e] Proportion mediated was defined as the proportion of the total effect mediated by adult loneliness (ACME divided by total effect).

© 2025 Wang J et al. *JAMA Network Open*.

**eTable 9. Associations between childhood loneliness and incident dementia during follow-up in the overall population and stratified by adult loneliness, with complete regression output for all covariates**

| Variable | Overall (n=13592) | | Adult non-loneliness group (n=8976) | | Adult loneliness group (n=3661) | |
|---|---|---|---|---|---|---|
| | HR (95% CI) [a] | P | HR (95% CI) [a] | P | HR (95% CI) [a] | P |
| **Model 1** | | | | | | |
| Childhood loneliness status | | | | | | |
| No childhood loneliness | 1 [Reference] | NA | 1 [Reference] | NA | 1 [Reference] | NA |
| Possible childhood loneliness | 0.91 (0.78-1.07) | .25 | 0.91 (0.74-1.13) | .40 | 1.04 (0.80-1.35) | .80 |
| Childhood loneliness | 1.41 (1.03-1.93) | .03 | 1.62 (1.04-2.51) | .03 | 1.38 (0.86-2.23) | .18 |
| Sex | | | | | | |
| Male | 1 [Reference] | NA | 1 [Reference] | NA | 1 [Reference] | NA |
| Female | 1.07 (0.91-1.25) | .40 | 1.14 (0.92-1.42) | .23 | 0.89 (0.69-1.15) | .36 |
| Childhood area of residence | | | | | | |
| Urban | 1 [Reference] | NA | 1 [Reference] | NA | 1 [Reference] | NA |
| Rural | 1.01 (0.77-1.33) | .93 | 1.09 (0.76-1.57) | .65 | 1.00 (0.62-1.61) | .99 |
| Educational level | | | | | | |
| No formal education | 1 [Reference] | NA | 1 [Reference] | NA | 1 [Reference] | NA |
| Junior high school or below | 1.24 (1.04-1.48) | .02 | 1.32 (1.05-1.67) | .02 | 1.35 (1.02-1.79) | .03 |
| High school or above | 1.26 (0.94-1.68) | .12 | 1.34 (0.92-1.95) | .12 | 1.77 (1.08-2.93) | .02 |
| Childhood socioeconomic position | | | | | | |
| High | 1 [Reference] | NA | 1 [Reference] | NA | 1 [Reference] | NA |
| Medium | 0.90 (0.69-1.18) | .45 | 0.99 (0.69-1.44) | .98 | 0.74 (0.48-1.12) | .15 |
| Low | 1.01 (0.77-1.33) | .92 | 1.19 (0.82-1.73) | .36 | 0.75 (0.49-1.16) | .20 |
| **Model 2** | | | | | | |
| Childhood loneliness status | | | | | | |
| No childhood loneliness | 1 [Reference] | NA | NA | NA | NA | NA |
| Possible childhood loneliness | 0.96 (0.81-1.13) | .61 | NA | NA | NA | NA |

© 2025 Wang J et al. *JAMA Network Open*.

| | | | | | | |
|---|---|---|---|---|---|---|
| Childhood loneliness | 1.46 (1.05-2.01) | .02 | NA | NA | NA | NA |
| Sex | | | | | | |
| Male | 1 [Reference] | NA | NA | NA | NA | NA |
| Female | 1.02 (0.87-1.21) | .80 | NA | NA | NA | NA |
| Childhood area of residence | | | | | | |
| Urban | 1 [Reference] | NA | NA | NA | NA | NA |
| Rural | 1.05 (0.78-1.40) | .76 | NA | NA | NA | NA |
| Educational level | | | | | | |
| No formal education | 1 [Reference] | NA | NA | NA | NA | NA |
| Junior high school or below | 1.34 (1.12-1.60) | .002 | NA | NA | NA | NA |
| High school or above | 1.45 (1.08-1.96) | .01 | NA | NA | NA | NA |
| Childhood socioeconomic position | | | | | | |
| High | 1 [Reference] | NA | NA | NA | NA | NA |
| Medium | 0.88 (0.67-1.16) | .35 | NA | NA | NA | NA |
| Low | 0.99 (0.74-1.31) | .92 | NA | NA | NA | NA |
| Adult loneliness | | | | | | |
| No adult loneliness | 1 [Reference] | NA | NA | NA | NA | NA |
| Adult loneliness | 1.63 (1.39-1.91) | <.001 | NA | NA | NA | NA |

Abbreviations: HR, hazard ratio; NA, not applicable.

[a] The HRs (95% CIs) were estimated using Cox proportional hazards regression models, with age as the timescale and left truncation by age at study entry. Model 1 was adjusted for age (time scale), sex, educational level, childhood area of residence, and childhood socioeconomic position. Model 2 was further adjusted for adult loneliness, which was included only among the 12,637 participants with available adult loneliness data. Missing data for other covariates were handled by multiple imputation with chained equations.

© 2025 Wang J et al. *JAMA Network Open*.

**eTable 10. Associations between two childhood loneliness items (often feel lonely in childhood; no close friends in childhood) and incident dementia during follow-up**

| Variable | Model 1 | | Model 2 | |
|---|---|---|---|---|
| | HR (95% CI) [a] | *P* | HR (95% CI) [a] | *P* |
| Childhood loneliness items | | | | |
| Often feel lonely in childhood | 1.50 (1.17-1.93) | .001 | 1.51 (1.17-1.95) | .002 |
| No close friends in childhood | 0.90 (0.77-1.05) | .19 | 0.94 (0.80-1.11) | .47 |

Abbreviations: HR, hazard ratio.

[a] The HRs (95% CIs) were estimated using Cox proportional hazards regression models, with age as the timescale and left truncation by age at study entry. Model 1 was adjusted for age (time scale), sex, educational level, childhood area of residence, and childhood socioeconomic position. Model 2 was further adjusted for adult loneliness, which was included only among the 12,637 participants with available adult loneliness data. Missing data for other covariates were handled by multiple imputation with chained equations. Models for each childhood-loneliness item were adjusted for the other item.

© 2025 Wang J et al. *JAMA Network Open*.

**eTable 11. Modifying role of adult loneliness in the associations between childhood loneliness and incident dementia during follow-up**

| Covariate | HR (95% CI) [a] | *P* |
|---|---|---|
| Childhood loneliness status | | |
| No childhood loneliness | 1 [Reference] | NA |
| Possible childhood loneliness | 0.94 (0.76-1.16) | .57 |
| Childhood loneliness | 1.71 (1.11-2.64) | .02 |
| Adult loneliness | 1.63 (1.28-2.08) | <.001 |
| Childhood loneliness status × adult loneliness | | |
| No childhood loneliness | 1 [Reference] | NA |
| Possible childhood loneliness | 1.04 (0.75-1.45) | .80 |
| Childhood loneliness | 0.73 (0.39-1.38) | .34 |
| Sex | | |
| Male | 1 [Reference] | NA |
| Female | 1.02 (0.87-1.21) | .79 |
| Childhood area of residence | | |
| Urban | 1 [Reference] | NA |
| Rural | 1.04 (0.78-1.39) | .78 |
| Educational level | | |
| No formal education | 1 [Reference] | NA |
| Junior high school or below | 1.34 (1.12-1.60) | .002 |
| High school or above | 1.45 (1.08-1.96) | .01 |
| Childhood socioeconomic position | | |
| High | 1 [Reference] | NA |
| Medium | 0.88 (0.67-1.16) | .36 |
| Low | 0.99 (0.75-1.31) | .93 |

Abbreviations: HR, hazard ratio; NA, not applicable.

[a] The HRs (95% CIs) were estimated using Cox proportional hazards regression models, with age as the timescale and left truncation by age at study entry. Model was adjusted for age (time scale), sex, educational level, childhood area of residence, and childhood socioeconomic position. A total of 12,637 participants with assessments of adult loneliness were included in adjusted models. Missing data for other covariates were handled by multiple imputation with chained equations.

© 2025 Wang J et al. *JAMA Network Open*.

**eTable 12. Joint associations of childhood and adulthood loneliness with cognitive decline and dementia risk during follow-up**

| Variables | Cognitive decline | | Incident dementia | |
|---|---|---|---|---|
| | *β* (95% CI) [a] | *P* | HR (95% CI) [a] | *P* |
| Loneliness status from childhood to adulthood | | | | |
| No childhood loneliness & no adult loneliness | 0 [Reference] | NA | 1 [Reference] | NA |
| Possible childhood loneliness & no adult loneliness | -0.06 (-0.10 to -0.03) | <.001 | 0.94 (0.76 to 1.16) | .57 |
| Childhood loneliness & no adult loneliness | -0.11 (-0.21 to -0.01) | .03 | 1.71 (1.11 to 2.64) | .02 |
| No childhood loneliness & adult loneliness | -0.17 (-0.22 to -0.12) | <.001 | 1.63 (1.28 to 2.08) | <.001 |
| Possible childhood loneliness & adult loneliness | -0.28 (-0.33 to -0.23) | <.001 | 1.60 (1.27 to 2.02) | <.001 |
| Childhood loneliness & adult loneliness | -0.28 (-0.40 to -0.17) | <.001 | 2.05 (1.30 to 3.22) | .002 |
| Time | -0.03 (-0.03 to -0.02) | <.001 | NA | NA |
| Loneliness status from childhood to adulthood × time | | | | |
| No childhood loneliness & no adult loneliness | 0 [Reference] | NA | NA | NA |
| Possible childhood loneliness & no adult loneliness | -0.02 (-0.02 to -0.01) | <.001 | NA | NA |
| Childhood loneliness & no adult loneliness | -0.04 (-0.06 to -0.02) | <.001 | NA | NA |
| No childhood loneliness & adult loneliness | 0.00 (-0.01 to 0.01) | .86 | NA | NA |
| Possible childhood loneliness & adult loneliness | -0.01 (-0.02 to -0.01) | <.001 | NA | NA |
| Childhood loneliness & adult loneliness | -0.03 (-0.05 to 0.00) | .03 | NA | NA |

Abbreviations: HR, hazard ratio; NA, not applicable.

[a] Models were adjusted for age, sex, educational level, childhood area of residence, and childhood socioeconomic position. Analyses were restricted to participants with available adult loneliness data (n = 10,834 for cognitive decline; n = 12,637 for dementia). Missing covariate data were addressed by multiple imputation with chained equations.

© 2025 Wang J et al. *JAMA Network Open*.

**eTable 13. Associations between childhood loneliness and cognitive decline during follow-up after excluding participants with missing data on covariates (n=10,806) [a]**

| Variable | Global cognitive function | | Episodic memory | | Executive function | |
|---|---|---|---|---|---|---|
| | *β* (95% CI) [b] | *P* | *β* (95% CI) [b] | *P* | *β* (95% CI) [b] | *P* |
| **Model 1** | | | | | | |
| Childhood loneliness status [c] | | | | | | |
| No childhood loneliness | 0 [Reference] | NA | 0 [Reference] | NA | 0 [Reference] | NA |
| Possible childhood loneliness | -0.08 (-0.11 to -0.04) | <.001 | -0.05 (-0.08 to -0.02) | .003 | -0.07 (-0.10 to -0.04) | <.001 |
| Childhood loneliness | -0.13 (-0.21 to -0.05) | .001 | 0.03 (-0.05 to 0.12) | .43 | -0.18 (-0.26 to -0.11) | <.001 |
| Time [d] | -0.03 (-0.03 to -0.02) | <.001 | 0.00 (0.00 to 0.01) | .48 | -0.04 (-0.04 to -0.03) | <.001 |
| Childhood loneliness status × time [d] | | | | | | |
| No childhood loneliness | 0 [Reference] | NA | 0 [Reference] | NA | 0 [Reference] | NA |
| Possible childhood loneliness | -0.02 (-0.02 to -0.01) | <.001 | -0.02 (-0.03 to -0.02) | <.001 | -0.01 (-0.01 to 0.00) | .003 |
| Childhood loneliness | -0.03 (-0.05 to -0.02) | <.001 | -0.05 (-0.07 to -0.03) | <.001 | -0.01 (-0.03 to 0.00) | .06 |
| **Model 2** | | | | | | |
| Childhood loneliness status [c] | | | | | | |
| No childhood loneliness | 0 [Reference] | NA | 0 [Reference] | NA | 0 [Reference] | NA |
| Possible childhood loneliness | -0.07 (-0.10 to -0.04) | <.001 | -0.05 (-0.08 to -0.01) | .005 | -0.07 (-0.10 to -0.04) | <.001 |
| Childhood loneliness | -0.10 (-0.18 to -0.02) | .01 | 0.05 (-0.03 to 0.14) | .24 | -0.16 (-0.24 to -0.08) | <.001 |
| Time [d] | -0.03 (-0.03 to -0.02) | <.001 | 0.00 (0.00 to 0.01) | .46 | -0.04 (-0.04 to -0.03) | <.001 |
| Childhood loneliness status × time [d] | | | | | | |
| No childhood loneliness | 0 [Reference] | NA | 0 [Reference] | NA | 0 [Reference] | NA |
| Possible childhood loneliness | -0.02 (-0.02 to -0.01) | <.001 | -0.02 (-0.03 to -0.02) | <.001 | -0.01 (-0.01 to 0.00) | .002 |
| Childhood loneliness | -0.03 (-0.05 to -0.02) | <.001 | -0.05 (-0.07 to -0.03) | <.001 | -0.01 (-0.03 to 0.00) | .06 |

Abbreviations: NA, not applicable.

[a] Of 10,880 participants, 74 with missing data on age, educational level, childhood area of residence, or childhood socioeconomic position were excluded, leaving 10,806 for analysis.

[b] The *β* coefficients were estimated using linear mixed-effects models. Model 1 was adjusted for age, sex, educational level, childhood area of residence, and childhood socioeconomic position. Model 2 was further adjusted for adult loneliness, which was included only among the 10,760 participants with available adult loneliness data.

© 2025 Wang J et al. *JAMA Network Open*.

[c] The *β* coefficient and its 95% CI are reported as SD.

[d] The *β* coefficient and its 95% CI are reported as SD per year.

© 2025 Wang J et al. *JAMA Network Open*.

**eTable 14. Associations between childhood loneliness and incident dementia during follow-up after excluding participants with missing data on covariates (n=13,461) [a]**

| Variable | Model 1 | | Model 2 | |
|---|---|---|---|---|
| | HR (95% CI) [b] | *P* | HR (95% CI) [b] | *P* |
| Childhood loneliness status | | | | |
| No childhood loneliness | 1 [Reference] | NA | 1 [Reference] | NA |
| Possible childhood loneliness | 0.92 (0.78-1.08) | .29 | 0.96 (0.81-1.13) | .63 |
| Childhood loneliness | 1.40 (1.02-1.92) | .04 | 1.43 (1.03-1.98) | .03 |

Abbreviations: HR, hazard ratio; NA, not applicable.

[a] Of 13,592 participants, 131 with missing data on age, educational level, childhood area of residence, or childhood socioeconomic position were excluded, leaving 13,461 for analysis.

[b] The HRs (95% CIs) were estimated using Cox proportional hazards regression models, with age as the timescale and left truncation by age at study entry. Model 1 was adjusted for age (time scale), sex, educational level, childhood area of residence, and childhood socioeconomic position. Model 2 was further adjusted for adult loneliness, which was included only among the 12,541 participants with available adult loneliness data.

© 2025 Wang J et al. *JAMA Network Open*.

**eTable 15. Associations between childhood loneliness and cognitive decline during follow-up after further adjustment for chronic diseases, depression, and healthy lifestyles (n=10,880)**

| Variable | Global cognitive function | | Episodic memory | | Executive function | |
|---|---|---|---|---|---|---|
| | $\beta$ (95% CI) [a] | *P* | $\beta$ (95% CI) [a] | *P* | $\beta$ (95% CI) [a] | *P* |
| **Model 1** | | | | | | |
| Childhood loneliness status [b] | | | | | | |
| No childhood loneliness | 0 [Reference] | NA | 0 [Reference] | NA | 0 [Reference] | NA |
| Possible childhood loneliness | -0.06 (-0.09 to -0.03) | <.001 | -0.04 (-0.07 to -0.01) | .01 | -0.06 (-0.09 to -0.03) | <.001 |
| Childhood loneliness | -0.08 (-0.16 to 0.00) | .04 | 0.07 (-0.01 to 0.16) | .10 | -0.14 (-0.22 to -0.07) | <.001 |
| Time [c] | -0.03 (-0.03 to -0.02) | <.001 | 0.00 (0.00 to 0.01) | .53 | -0.04 (-0.04 to -0.03) | <.001 |
| Childhood loneliness status × time [c] | | | | | | |
| No childhood loneliness | 0 [Reference] | | 0 [Reference] | | 0 [Reference] | |
| Possible childhood loneliness | -0.02 (-0.02 to -0.01) | <.001 | -0.02 (-0.03 to -0.02) | <.001 | -0.01 (-0.01 to 0.00) | .004 |
| Childhood loneliness | -0.03 (-0.05 to -0.02) | <.001 | -0.05 (-0.07 to -0.03) | <.001 | -0.01 (-0.03 to 0.00) | .06 |
| **Model 2** | | | | | | |
| Childhood loneliness status [b] | | | | | | |
| No childhood loneliness | 0 [Reference] | NA | 0 [Reference] | NA | 0 [Reference] | NA |
| Possible childhood loneliness | -0.06 (-0.09 to -0.03) | <.001 | -0.04 (-0.07 to -0.01) | .01 | -0.06 (-0.09 to -0.03) | <.001 |
| Childhood loneliness | -0.08 (-0.15 to 0.00) | .05 | 0.07 (-0.01 to 0.16) | .09 | -0.14 (-0.22 to -0.06) | <.001 |
| Time [c] | -0.03 (-0.03 to -0.02) | <.001 | 0.00 (0.00 to 0.01) | .50 | -0.04 (-0.04 to -0.03) | <.001 |
| Childhood loneliness status × time [c] | | | | | | |
| No childhood loneliness | 0 [Reference] | NA | 0 [Reference] | NA | 0 [Reference] | NA |
| Possible childhood loneliness | -0.02 (-0.02 to -0.01) | <.001 | -0.02 (-0.03 to -0.02) | <.001 | -0.01 (-0.01 to 0.00) | .003 |
| Childhood loneliness | -0.03 (-0.05 to -0.02) | <.001 | -0.05 (-0.07 to -0.03) | <.001 | -0.01 (-0.03 to 0.00) | .06 |

Abbreviations: NA, not applicable.

[a] The $\beta$ coefficients were estimated using linear mixed-effects models. Model 1 was adjusted for age, sex, educational level, childhood area of residence, childhood socioeconomic position, heart diseases, stroke, cancer, diabetes, depression score, smoking status, alcohol consumption, and sleep duration. Model 2 was further adjusted for adult loneliness, which was included only among the 10,834 participants with available adult loneliness data. Missing data for other covariates (demographic characteristics, chronic diseases, depression score, and healthy

© 2025 Wang J et al. *JAMA Network Open*.

lifestyle factors) were handled by multiple imputation with chained equations. Variance inflation factors for all covariates ranged from 1.01 (cancer) to 2.25 (sex), indicating no evidence of multicollinearity.

[b] The *β* coefficient and its 95% CI are reported as SD.

[c] The *β* coefficient and its 95% CI are reported as SD per year.

© 2025 Wang J et al. *JAMA Network Open*.

## eTable 16. Associations between childhood loneliness and incident dementia during follow-up after further adjustment for chronic diseases, depression, and healthy lifestyles (n=13,592)

| Variable | Model 1 | | Model 2 | |
|---|---|---|---|---|
| | HR (95% CI) [a] | *P* | HR (95% CI) [a] | *P* |
| Childhood loneliness status | | | | |
| No childhood loneliness | 1 [Reference] | NA | 1 [Reference] | NA |
| Possible childhood loneliness | 0.90 (0.77-1.06) | .19 | 0.94 (0.80-1.11) | .50 |
| Childhood loneliness | 1.39 (1.02-1.90) | .04 | 1.47 (1.06-2.02) | .02 |

Abbreviations: HR, hazard ratio; NA, not applicable.

[a] The HRs (95% CIs) were estimated using Cox proportional hazards regression models, with age as the timescale and left truncation by age at study entry. Model 1 was adjusted for age (time scale), sex, educational level, childhood area of residence, childhood socioeconomic position, heart diseases, stroke, cancer, diabetes, depression score, smoking status, alcohol consumption, and sleep duration. Model 2 was further adjusted for adult loneliness, which was included only among the 12,637 participants with available adult loneliness data. Missing data for other covariates (demographic characteristics, chronic diseases, depression score, and healthy lifestyle factors) were handled by multiple imputation with chained equations. Variance inflation factors for all covariates ranged from 1.01 (cancer) to 2.02 (sex), indicating no evidence of multicollinearity.

© 2025 Wang J et al. *JAMA Network Open*.

**eTable 17. Associations between childhood loneliness and cognitive decline during follow-up after excluding participants with cognitive impairment at baseline (n=10,244) [a]**

| Variable | Global cognitive function | | Episodic memory | | Executive function | |
|---|---|---|---|---|---|---|
| | *β* (95% CI) [b] | *P* | *β* (95% CI) [b] | *P* | *β* (95% CI) [b] | *P* |
| **Model 1** | | | | | | |
| Childhood loneliness status [c] | | | | | | |
| No childhood loneliness | 0 [Reference] | NA | 0 [Reference] | NA | 0 [Reference] | NA |
| Possible childhood loneliness | -0.07 (-0.10 to -0.04) | <.001 | -0.05 (-0.08 to -0.01) | .006 | -0.06 (-0.09 to -0.03) | <.001 |
| Childhood loneliness | -0.12 (-0.20 to -0.05) | <.001 | 0.04 (-0.05 to 0.13) | .35 | -0.17 (-0.25 to -0.10) | <.001 |
| Time [d] | -0.04 (-0.04 to -0.03) | <.001 | 0.00 (-0.01 to 0.00) | .12 | -0.04 (-0.05 to -0.04) | <.001 |
| Childhood loneliness status × time [d] | | | | | | |
| No childhood loneliness | 0 [Reference] | NA | 0 [Reference] | NA | 0 [Reference] | NA |
| Possible childhood loneliness | -0.02 (-0.02 to -0.01) | <.001 | -0.02 (-0.03 to -0.01) | <.001 | -0.01 (-0.01 to 0.00) | .003 |
| Childhood loneliness | -0.04 (-0.05 to -0.02) | <.001 | -0.05 (-0.07 to -0.03) | <.001 | -0.02 (-0.03 to 0.00) | .01 |
| **Model 2** | | | | | | |
| Childhood loneliness status [c] | | | | | | |
| No childhood loneliness | 0 [Reference] | NA | 0 [Reference] | NA | 0 [Reference] | NA |
| Possible childhood loneliness | -0.07 (-0.10 to -0.04) | <.001 | -0.04 (-0.08 to -0.01) | .009 | -0.06 (-0.09 to -0.03) | <.001 |
| Childhood loneliness | -0.09 (-0.17 to -0.02) | .01 | 0.06 (-0.03 to 0.15) | .19 | -0.15 (-0.22 to -0.07) | <.001 |
| Time [d] | -0.04 (-0.04 to -0.03) | <.001 | 0.00 (-0.01 to 0.00) | .13 | -0.04 (-0.05 to -0.04) | <.001 |
| Childhood loneliness status × time [d] | | | | | | |
| No childhood loneliness | 0 [Reference] | NA | 0 [Reference] | NA | 0 [Reference] | NA |
| Possible childhood loneliness | -0.02 (-0.02 to -0.01) | <.001 | -0.02 (-0.03 to -0.02) | <.001 | -0.01 (-0.01 to 0.00) | .003 |
| Childhood loneliness | -0.04 (-0.05 to -0.02) | <.001 | -0.05 (-0.07 to -0.03) | <.001 | -0.02 (-0.03 to 0.00) | .01 |

Abbreviations: NA, not applicable.

[a] Of 10,880 participants without dementia (defined as the coexistence of cognitive impairment and functional impairment, or a physician's diagnosis of dementia) at baseline, an additional 636 participants classified as having cognitive impairment were excluded, leaving 10,244 for analysis.

[b] The *β* coefficients were estimated using linear mixed-effects models. Model 1 was adjusted for age, sex, educational level, childhood area of residence, and childhood socioeconomic

© 2025 Wang J et al. *JAMA Network Open*.

position. Model 2 was further adjusted for adult loneliness, which was included only among the 10,205 participants with available adult loneliness data. Missing data for other covariates were handled by multiple imputation with chained equations.

[c] The *β* coefficient and its 95% CI are reported as SD.

[d] The *β* coefficient and its 95% CI are reported as SD per year.

© 2025 Wang J et al. *JAMA Network Open*.

**eTable 18. Associations between childhood loneliness and incident dementia during follow-up after excluding participants with cognitive impairment at baseline (n=12,883) [a]**

| Variable | Model 1 | | Model 2 | |
|---|---|---|---|---|
| | HR (95% CI) [b] | *P* | HR (95% CI) [b] | *P* |
| Childhood loneliness status | | | | |
| No childhood loneliness | 1 [Reference] | NA | 1 [Reference] | NA |
| Possible childhood loneliness | 0.92 (0.78-1.08) | .29 | 0.96 (0.81-1.13) | .63 |
| Childhood loneliness | 1.40 (1.02-1.92) | .04 | 1.43 (1.03-1.98) | .03 |

Abbreviations: HR, hazard ratio; NA, not applicable.

[a] Of 13,592 participants without dementia (defined as the coexistence of cognitive impairment and functional impairment, or a physician's diagnosis of dementia) at baseline, an additional 709 participants classified as having cognitive impairment were excluded, leaving 12,883 for analysis.

[b] The HRs (95% CIs) were estimated using Cox proportional hazards regression models, with age as the timescale and left truncation by age at study entry. Model 1 was adjusted for age (time scale), sex, educational level, childhood area of residence, and childhood socioeconomic position. Model 2 was further adjusted for adult loneliness, which was included only among the 11,938 participants with available adult loneliness data. Missing data for other covariates were handled by multiple imputation with chained equations.

© 2025 Wang J et al. *JAMA Network Open*.

**eTable 19. Associations between frequency of childhood loneliness and cognitive decline during follow-up (n=10,880)**

| Variable | Global cognitive function | | Episodic memory | | Executive function | |
|---|---|---|---|---|---|---|
| | *β* (95% CI) [a] | *P* | *β* (95% CI) [a] | *P* | *β* (95% CI) [a] | *P* |
| **Model 1** | | | | | | |
| Frequency of childhood loneliness [b] | | | | | | |
| Never [d] | 0 [Reference] | NA | 0 [Reference] | NA | 0 [Reference] | NA |
| Rarely [d] | -0.07 (-0.12 to -0.01) | .01 | -0.02 (-0.07 to 0.04) | .61 | -0.08 (-0.13 to -0.03) | .004 |
| Sometimes [d] | -0.05 (-0.11 to 0.01) | .14 | -0.04 (-0.11 to 0.03) | .23 | -0.04 (-0.10 to 0.02) | .22 |
| Often [d] | -0.10 (-0.16 to -0.04) | .002 | 0.03 (-0.04 to 0.10) | .38 | -0.14 (-0.21 to -0.08) | <.001 |
| *P* value for trend [e] | NA | <.001 | NA | .98 | NA | <.001 |
| Time [c] | -0.03 (-0.04 to -0.03) | <.001 | -0.01 (-0.01 to 0.00) | .001 | -0.04 (-0.04 to -0.04) | <.001 |
| Frequency of childhood loneliness × time [c] | | | | | | |
| Never [d] | 0 [Reference] | NA | 0 [Reference] | NA | 0 [Reference] | NA |
| Rarely [d] | -0.01 (-0.02 to 0.00) | .12 | -0.01 (-0.03 to 0.00) | .05 | 0.00 (-0.01 to 0.01) | .49 |
| Sometimes [d] | -0.01 (-0.02 to 0.00) | .04 | -0.01 (-0.02 to 0.01) | .34 | -0.01 (-0.02 to 0.00) | .03 |
| Often [d] | -0.02 (-0.03 to -0.01) | <.001 | -0.03 (-0.05 to -0.02) | <.001 | -0.01 (-0.02 to 0.00) | .07 |
| *P* value for trend [e] | NA | <.001 | NA | <.001 | NA | .008 |
| **Model 2** | | | | | | |
| Frequency of childhood loneliness [b] | | | | | | |
| Never [d] | 0 [Reference] | NA | 0 [Reference] | NA | 0 [Reference] | NA |
| Rarely [d] | -0.05 (-0.11 to 0.00) | .05 | -0.01 (-0.06 to 0.05) | .86 | -0.07 (-0.12 to -0.01) | .01 |
| Sometimes [d] | -0.03 (-0.09 to 0.03) | .40 | -0.03 (-0.09 to 0.04) | .42 | -0.02 (-0.08 to 0.04) | .53 |
| Often [d] | -0.07 (-0.13 to -0.01) | .02 | 0.05 (-0.02 to 0.12) | .16 | -0.12 (-0.18 to -0.06) | <.001 |
| *P* value for trend [e] | NA | .009 | NA | .51 | NA | <.001 |
| Time [c] | -0.03 (-0.04 to -0.03) | <.001 | -0.01 (-0.01 to 0.00) | .001 | -0.04 (-0.04 to -0.04) | <.001 |
| Frequency of childhood loneliness × time [c] | | | | | | |
| Never [d] | 0 [Reference] | NA | 0 [Reference] | NA | 0 [Reference] | NA |

© 2025 Wang J et al. *JAMA Network Open*.

| | | | | | | |
|---|---|---|---|---|---|---|
| Rarely [d] | -0.01 (-0.02 to 0.00) | .12 | -0.01 (-0.03 to 0.00) | 0.04 | 0.00 (-0.01 to 0.01) | .50 |
| Sometimes [d] | -0.01 (-0.02 to 0.00) | .03 | -0.01 (-0.02 to 0.01) | 0.32 | -0.01 (-0.02 to 0.00) | .03 |
| Often [d] | -0.02 (-0.03 to -0.01) | <.001 | -0.03 (-0.05 to -0.02) | <.001 | -0.01 (-0.02 to 0.00) | .07 |
| *P* value for trend [e] | NA | <.001 | NA | <.001 | NA | .008 |

Abbreviations: NA, not applicable.

[a] The $\beta$ coefficients were estimated using linear mixed-effects models. Model 1 was adjusted for age, sex, educational level, childhood area of residence, and childhood socioeconomic position. Model 2 was further adjusted for adult loneliness, which was included only among the 10,834 participants with available adult loneliness data. Missing data for other covariates were handled by multiple imputation with chained equations.

[b] The $\beta$ coefficient and its 95% CI are reported as SD.

[c] The $\beta$ coefficient and its 95% CI are reported as SD per year.

[d] Frequency of childhood loneliness was categorized as follows: never (n = 8,629), rarely (n = 887), sometimes (n = 683), and often (n = 681).

[e] *P* value for trend was calculated using the frequency of childhood loneliness as a continuous variable.

## eTable 20. Associations between frequency of childhood loneliness and incident dementia during follow-up (n=13,592)

| Variable | N | Cases (incidence rate per 1000 person-years) | Model 1 | | Model 2 | |
|---|---|---|---|---|---|---|
| | | | HR (95% CI) [a] | *P* | HR (95% CI) [a] | *P* |
| Frequency of childhood loneliness | | | | | | |
| Never | 10702 | 520 (7.53) | 1 [Reference] | NA | 1 [Reference] | NA |
| Rarely | 1136 | 61 (8.37) | 1.05 (0.81-1.38) | .70 | 1.03 (0.78-1.36) | .85 |
| Sometimes | 871 | 45 (8.01) | 1.05 (0.77-1.43) | .76 | 0.91 (0.65-1.27) | .58 |
| Often | 883 | 71 (12.74) | 1.51 (1.17-1.94) | .002 | 1.49 (1.15-1.93) | .002 |
| *P* value for trend [b] | NA | NA | NA | .006 | NA | .03 |

Abbreviations: HR, hazard ratio; NA, not applicable.

[a] The HRs (95% CIs) were estimated using Cox proportional hazards regression models, with age as the timescale and left truncation by age at study entry. Model 1 was adjusted for age (time scale), sex, educational level, childhood area of residence, and childhood socioeconomic position. Model 2 was further adjusted for adult loneliness, which was included only among the 12,637 participants with available adult loneliness data. Missing data for other covariates were handled by multiple imputation with chained equations.

[b] *P* value for trend was calculated using the frequency of childhood loneliness as a continuous variable.

© 2025 Wang J et al. *JAMA Network Open*.

## eTable 21. Associations of childhood loneliness with executive function, assessed using mixed-effects Tobit models to account for ceiling effects (n=10,880)

| Variable | Model 1 | | Model 2 | |
|---|---|---|---|---|
| | *β* (95% CI) [a] | *P* | *β* (95% CI) [a] | *P* |
| Childhood loneliness status [b] | | | | |
| No childhood loneliness | 0 [Reference] | NA | 0 [Reference] | NA |
| Possible childhood loneliness | -0.10 (-0.13 to -0.06) | <.001 | -0.09 (-0.13 to -0.06) | <.001 |
| Childhood loneliness | -0.25 (-0.34 to -0.16) | <.001 | -0.22 (-0.31 to -0.13) | <.001 |
| Time [c] | -0.05 (-0.05 to -0.04) | <.001 | -0.05 (-0.05 to -0.04) | <.001 |
| Childhood loneliness status × time [c] | | | | |
| No childhood loneliness | 0 [Reference] | NA | 0 [Reference] | NA |
| Possible childhood loneliness | 0.00 (−0.01 to 0.00) | .20 | 0.00 (-0.01 to 0.00) | .17 |
| Childhood loneliness | 0.00 (-0.02 to 0.01) | .63 | 0.00 (-0.02 to 0.01) | .63 |
| Age [c] | -0.01 (-0.01 to -0.01) | <.001 | -0.01 (-0.01 to -0.01) | <.001 |
| Sex [b] | | | | |
| Male | 0 [Reference] | NA | 0 [Reference] | NA |
| Female | -0.34 (-0.38 to -0.31) | <.001 | -0.33 (-0.36 to -0.30) | <.001 |
| Educational level [b] | | | | |
| No formal education | 0 [Reference] | NA | 0 [Reference] | NA |
| Junior high school or below | 0.91 (0.88 to 0.95) | <.001 | 0.90 (0.86 to 0.93) | <.001 |
| High school or above | 1.29 (1.23 to 1.34) | <.001 | 1.26 (1.21 to 1.31) | <.001 |
| Childhood area of residence [b] | | | | |
| Urban | 0 [Reference] | NA | 0 [Reference] | NA |
| Rural | -0.36 (-0.42 to -0.31) | <.001 | -0.35 (-0.41 to -0.30) | <.001 |
| Childhood socioeconomic position [b] | | | | |
| High | 0 [Reference] | NA | 0 [Reference] | NA |
| Medium | -0.01 (-0.07 to 0.04) | .68 | -0.01 (-0.06 to 0.05) | .78 |
| Low | -0.09 (-0.14 to -0.03) | 0.003 | -0.08 (-0.13 to -0.02) | .008 |
| Adult loneliness [b] | | | | |
| No adult loneliness | NA | NA | 0 [Reference] | NA |
| Adult loneliness | NA | NA | -0.21 (-0.25 to -0.18) | <.001 |

Abbreviations: NA, not applicable.

[a] The *β* coefficient was estimated using mixed-effects Tobit models. Model 1 was adjusted for age, sex, educational level, childhood area of residence, and childhood socioeconomic position. Model 2 was further adjusted for adult loneliness, which was included only among the 10,834 participants with available adult loneliness data. Missing data for other covariates were handled by multiple imputation with chained equations.

[b] The *β* coefficient and its 95% CI are reported as SD.

[c] The *β* coefficient and its 95% CI are reported as SD per year.

© 2025 Wang J et al. *JAMA Network Open.*

## eReferences


1. Amieva H, Retuerto N, Hernandez-Ruiz V, Meillon C, Dartigues JF, Pérès K. Longitudinal Study of Cognitive Decline before and after the COVID-19 Pandemic: Evidence from the PA-COVID Survey. *Dement Geriatr Cogn Disord.* 2022;51(1):56-62.
2. Chen S, Chen X, Hou X, Fang H, Liu GG, Yan LL. Temporal trends and disparities of population attributable fractions of modifiable risk factors for dementia in China: a time-series study of the China health and retirement longitudinal study (2011-2018). *Lancet Reg Health West Pac.* 2024;47:101106.
3. Xie X, Que J, Sun L, Sun T, Yang F. Association between urbanization levels and frailty among middle-aged and older adults in China: evidence from the CHARLS. *BMC Med.* 2025;23(1):171.
4. Angold A, Costello EJ. The Child and Adolescent Psychiatric Assessment (CAPA). *J Am Acad Child Adolesc Psychiatry.* 2000;39(1):39-48.
5. Xerxa Y, Rescorla LA, Shanahan L, Tiemeier H, Copeland WE. Childhood loneliness as a specific risk factor for adult psychiatric disorders. *Psychol Med.* 2023;53(1):227-235.
6. Elovainio M, Komulainen K, Sipilä PN, et al. Association of social isolation and loneliness with risk of incident hospital-treated infections: an analysis of data from the UK Biobank and Finnish Health and Social Support studies. *Lancet Public Health.* 2023;8(2):e109-e118.
7. Song Y, Zhu C, Shi B, et al. Social isolation, loneliness, and incident type 2 diabetes mellitus: results from two large prospective cohorts in Europe and East Asia and Mendelian randomization. *EClinicalMedicine.* 2023;64:102236.
8. Liang YY, Chen Y, Feng H, et al. Association of Social Isolation and Loneliness With Incident Heart Failure in a Population-Based Cohort Study. *JACC Heart Fail.* 2023;11(3):334-344.
9. Wang X, Ma H, Li X, Heianza Y, Fonseca V, Qi L. Joint association of loneliness and traditional risk factor control and incident cardiovascular disease in diabetes patients. *Eur Heart J.* 2023;44(28):2583-2591.
10. Guo L, An L, Luo F, Yu B. Social isolation, loneliness and functional disability in Chinese older women and men: a longitudinal study. *Age and Ageing.* 2020;50(4):1222-1228.
11. Akhter-Khan SC, Tao Q, Ang TFA, et al. Associations of loneliness with risk of Alzheimer's disease dementia in the Framingham Heart Study. *Alzheimers Dement.* 2021;17(10):1619-1627.
12. Tao Q, Akhter-Khan SC, Ang TFA, et al. Different loneliness types, cognitive function, and brain structure in midlife: Findings from the Framingham Heart Study. *EClinicalMedicine.* 2022;53:101643.
13. Wang J, Jin R, Wu Z, et al. Moderate increase of serum uric acid within a normal range is associated with improved cognitive function in a non-normotensive population: A nationally representative cohort study. *Front Aging Neurosci.* 2022;14:944341.
14. Wang J, Liu Y, Jin R, et al. Intraindividual difference in estimated GFR by creatinine and cystatin C, cognitive trajectories and motoric cognitive risk syndrome. *Nephrol Dial Transplant.* 2024;39(5):860-872.
15. Lin L, Cao B, Chen W, Li J, Zhang Y, Guo VY. Association of Adverse Childhood Experiences and Social Isolation With Later-Life Cognitive Function Among Adults in China. *JAMA Netw Open.* 2022;5(11):e2241714.
16. Liu Y, Gao X, Zhang Y, et al. Geographical variation in dementia prevalence across China: a geospatial analysis. *Lancet Reg Health West Pac.* 2024;47:101117.
17. Ahmadi-Abhari S, Guzman-Castillo M, Bandosz P, et al. Temporal trend in dementia incidence since 2002 and projections for prevalence in England and Wales to 2040: modelling study. *Bmj.* 2017;358:j2856.
18. Liu Y, Wu Y, Cai J, et al. Is there a common latent cognitive construct for dementia estimation across two Chinese cohorts? *Alzheimers Dement (Amst).* 2022;14(1):e12356.
19. Yang JJ, Yu D, Wen W, et al. Association of Diabetes With All-Cause and Cause-Specific Mortality in Asia: A Pooled Analysis of More Than 1 Million Participants. JAMA Netw Open. 2019;2(4):e192696.
20. Shi B, Choirat C, Coull BA, VanderWeele TJ, Valeri L. CMAverse: A Suite of Functions for Reproducible

Causal Mediation Analyses. *Epidemiology.* 2021;32(5):e20-e22.

21. van der Heide FCT, Valeri L, Dugravot A, et al. Role of cardiovascular health factors in mediating social inequalities in the incidence of dementia in the UK: two prospective, population-based cohort studies. *EClinicalMedicine.* 2024;70:102539.

22. Luo S, Chen W, Hu W, et al. Parental Education, Own Education, and Cognitive Function in Middle-Aged and Older Adults. *JAMA Netw Open.* 2025;8(5):e2513036.

© 2025 Wang J et al. *JAMA Network Open.*
