# Supplementary material for: Childhood Loneliness and Cognitive Decline and Dementia Risk in Middle-Aged and Older Adults
Source: JAMA Netw Open. 2025 Sep 12;8(9):e2531493. doi: 10.1001/jamanetworkopen.2025.31493 (PMC12432641; doi:10.1001/jamanetworkopen.2025.31493)
Supplement: Supplement 2. — Data Sharing Statement [file jamanetwopen-e2531493-s002.pdf]

# Data Sharing Statement

Wang. Childhood Loneliness and Cognitive Decline and Dementia Risk in Middle-Aged and Older Adults. *JAMA Netw Open*. Published September 12, 2025.
doi:10.1001/jamanetworkopen.2025.31493

**Data**
**Data available:** Yes
**Data types:** Deidentified participant data
**How to access data:** Materials are available upon request to the corresponding author (statguo@ccmu.edu.cn).
**When available:** With publication

**Supporting Documents**
**Document types:** None

**Additional Information**
**Who can access the data:** researchers whose proposed use of the data has been approved
**Types of analyses:** for any purpose or for a specified purpose
**Mechanisms of data availability:** after approval of a proposal
